# Universal control of four singlet–triplet qubits

**DOI:** 10.1038/s41565-024-01817-9

**Published:** 2024-10-31

**Authors:** Xin Zhang, Elizaveta Morozova, Maximilian Rimbach-Russ, Daniel Jirovec, Tzu-Kan Hsiao, Pablo Cova Fariña, Chien-An Wang, Stefan D. Oosterhout, Amir Sammak, Giordano Scappucci, Menno Veldhorst, Lieven M. K. Vandersypen

**Affiliations:** 1 QuTech, Delft University of Technology, Delft, Netherlands; 2 Kavli Institute of Nanoscience, Delft University of Technology, Delft, Netherlands; 3 Netherlands Organisation for Applied Scientific Research (TNO), Delft, Netherlands

**Keywords:** Quantum information, Electronic devices

## Abstract

The coherent control of interacting spins in semiconductor quantum dots is of strong interest for quantum information processing and for studying quantum magnetism from the bottom up. Here we present a 2 × 4 germanium quantum dot array with full and controllable interactions between nearest-neighbour spins. As a demonstration of the level of control, we define four singlet–triplet qubits in this system and show two-axis single-qubit control of each qubit and SWAP-style two-qubit gates between all neighbouring qubit pairs, yielding average single-qubit gate fidelities of 99.49(8)–99.84(1)% and Bell state fidelities of 73(1)–90(1)%. Combining these operations, we experimentally implement a circuit designed to generate and distribute entanglement across the array. A remote Bell state with a fidelity of 75(2)% and concurrence of 22(4)% is achieved. These results highlight the potential of singlet–triplet qubits as a competing platform for quantum computing and indicate that scaling up the control of quantum dot spins in extended bilinear arrays can be feasible.

## Abstract (web summary)

Singlet–triplet qubits implemented in a 2 × 4 germanium quantum dot array allow for a quantum circuit that generates and distributes entanglement across the array with a remote Bell state fidelity of 75(2)% between the first and last qubit.

## Main

The coherent control of a large-scale array of spins in the solid state represents a major challenge in the field of quantum-coherent nanoscience^1–4^. As a quintessential platform for quantum spin control, the lithographically defined semiconductor quantum dot has shown great promise both for fault-tolerant digital quantum computation^5–9^ and for analogue quantum simulation of emergent quantum phenomena^10–13^. Nevertheless, the inherent nanoscale dimensions of the devices, the geometric constraints in integrating all the required components, and the necessity of using high-frequency electromagnetic fields in cryogenic environments present important challenges for the integration and control of a large number of spins.

Significant efforts have already been undertaken to tackle these challenges. For single-spin qubits, the number of coherently controlled interacting spins has been scaled up to six in a one-dimensional array^14^ and to four in a two-dimensional array^15^. A six-dot linear array was also used to achieve universal control of two qubits that are each encoded in a subspace of three electron spins distributed over three dots^16^. For singlet–triplet qubits defined in a subspace of two spins across two dots, recent progress includes the individual control of three to four uncoupled qubits^17,18^ and the operation of a single qubit in a 3 × 3 quantum dot array^19^.

Similar to exchange-only qubits, singlet–triplet qubits^20–24^ allow fully electrical qubit control using baseband voltage pulses. The use of baseband-only control signals can avoid commonly encountered problems of single-spin qubits such as microwave heating effects^14,25,26^ and may furthermore alleviate crosstalk effects^27^. Singlet–triplet qubits also map naturally to the spin-readout basis in Pauli spin blockade (PSB), a common method for spin-to-charge conversion in quantum dots^21,28^. By using pulse optimization, single-qubit control fidelities of singlet–triplet qubits have exceeded 99%^29^, whereas two-qubit gate fidelities relying on the relatively weak capacitive (Coulomb) interaction reached 72–90%^30,31^. In theory, the two-qubit gate fidelity can be further improved by replacing the capacitive coupling with the stronger exchange coupling^32^ although this has been little investigated in experiments^33^. Despite this progress, universal control of more than two interacting singlet–triplet qubits has yet to be achieved. Recently, a controlled number of charge carriers were loaded in 2 × 4 arrays, a 4 × 4 array and a 1 × 12 array^34–38^. These advances set the stage for exploring the operation of three or more interacting singlet–triplet qubits experimentally.

Here we demonstrate coherent control of four interacting singlet–triplet qubits in a 2 × 4 germanium quantum dot array, which forms a quantum dot ladder. Taking advantage of the strong intrinsic spin–orbit coupling and small in-plane *g*-factors of holes in strained germanium quantum wells^39^, we encode the qubit in the singlet ($\left\vert {\mathrm{S}} \right\rangle$) and the lowest triplet ($\left\vert {\mathrm{T}}_{-}\right\rangle$) of two exchange-coupled spins, a variant of the originally proposed singlet–triplet qubit^40–44^. By controlling the exchange interaction inside each spin pair along the ladder rungs, we first map out the qubit energy spectrum. Then we show universal control of each qubit by pulsing both the detuning and tunnelling barrier of the corresponding double quantum dot (DQD). With proper simultaneous control of detunings and tunnelling barriers of neighbouring S–T_−_ qubits, we achieve a two-qubit SWAP-style gate, a quantum gate that swaps the information of two qubits, induced by exchange interactions for each pair of neighbouring qubits in the ladder. Finally, with the demonstrated single- and two-qubit control, we implement a quantum circuit for quantum state transfer across the ladder.

## Germanium quantum dot ladder

**Fig. 1:**
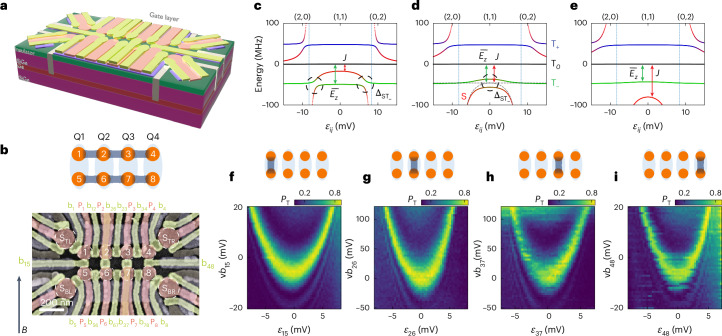
Device and energy spectroscopy. **a**, Schematic drawing showing the Ge/SiGe heterostructure and three layers of gate electrodes on top to define the quantum dot ladder and sensing dots: screening gates (purple), plunger gates (red) and barrier gates (green). Ohmic contacts (grey) extend towards the germanium quantum well in which the holes are confined. The aluminium oxide dielectric between different gate layers is omitted for clarity. **b**, False-coloured scanning electron microscope image of a device nominally identical to that used in the measurements. The eight quantum dots are labelled 1–8 and the four charge sensors to measure the charge states in the quantum dots are labelled S_TL_, S_TR_, S_BL_ and S_BR_, respectively. The quantum dot potentials are controlled by plunger gates P_*i*_, and the interdot or dot–sensor tunnel couplings are controlled by barrier gates b_*ij*_ or b_*i*_, with *i* or *j* denoting the corresponding quantum dot number. A schematic of the ladder structure of the quantum dots is shown on top, with Q1–Q4 formed by vertical DQDs. **c**–**e**, The energy levels of two-spin states in a DQD as a function of energy detuning *ε*_*ij*_ between dot *i* and *j* with $J({\varepsilon }_{ij}=0) < {\overline{E}}_{z}\,({\bf{c}}),J({\varepsilon }_{ij}=0)={\overline{E}}_{z}\,({\bf{d}}),J({\varepsilon }_{ij}=0) > {\overline{E}}_{z} ({\bf{e}})$. The dashed black circles denote the positions of S–T_−_ anticrossings. **f**–**i**, The measured energy spectra that probe the positions of the S–T_−_ anticrossings as a function of the detuning (*ε*_*ij*_) and the barrier gate voltage (v*b*_*ij*_) for the vertical DQDs 1–5 (**f**), 2–6 (**g**), 3–7 (**h**) and 4–8 (**i**) at *B* = 5 mT. The colour scale shows the measured spin triplet probability *P*_T_ after initializing a vertical DQD in a singlet state (in (0,2) or (2,0)) and applying a gate voltage pulse (20 ns ramp in, 50 or 60 ns wait time, 0 ns ramp out to the PSB regime for readout) to the detuning shown on the horizontal axis, for different v*b*_*ij*_. The 20 ns ramp-in time is used to ensure adiabaticity with respect to the tunnel coupling (2 GHz), while maintaining diabaticity relative to the S–T_−_ anticrossing. The wait time is close to a π rotation to obtain a sizable triplet probability. The cartoons on top of the panels **f**–**i** represent the eight dots, and the dark grey line indicates which exchange coupling is active in the panel below.

**Extended Data Fig. 1:**
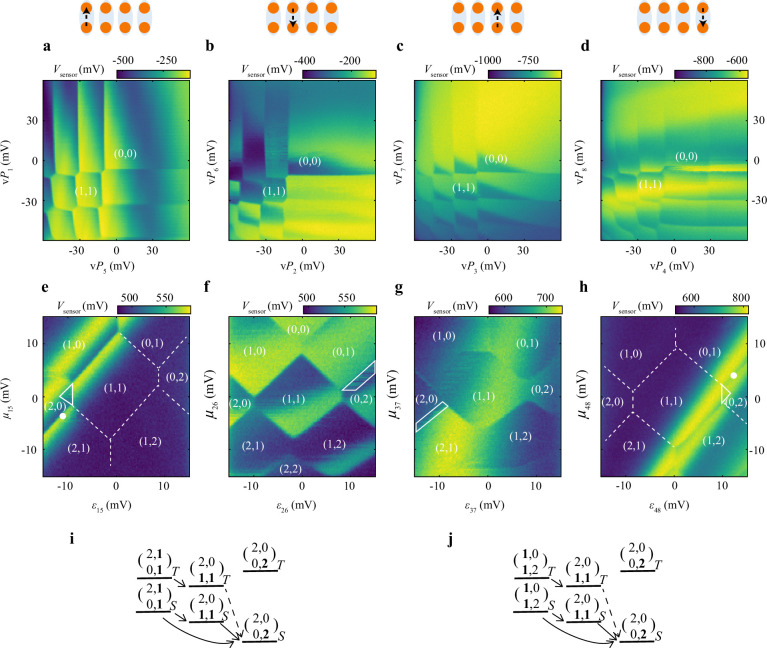
Charge stability diagrams and Pauli spin blockade. **a-d**, Charge stability diagrams for DQD 1-5 (**a**), 2-6 (**b**), 3-7 (**c**), and 4-8 (**d**), respectively. **a** and **b** are recorded using the sensor S_BL_ while **c** and **d** are recorded using the sensor S_BR_. Hole numbers inside the relevant charge stability regions are indicated, showing all the DQDs can be emptied to (0,0). **e-h**, Charge stability diagrams measured by scanning the detuning *ε*_*ij*_ and the overall chemical potential *μ*_*ij*_ of the DQD. The PSB regions inside the (2, 0) or (0, 2) area are indicated by solid white triangles and trapezoids. For outer DQD 1-5 and 4-8, we find PSB by pulsing *ε*_15_ and *ε*_48_ from (1,1) to (2,0) and (0,2), where within a triangular region an electron tunnels between the dots starting from the *S*(1, 1) but no tunnelling occurs from *T*_0_(1, 1), *T*_−_(1, 1) and *T*_+_(1, 1) due to PSB. For inner DQD 2-6 and 3-7, we swap their spin states to those of DQD 5-6 and 7-8 where the sensor signals are stronger. **i**,**j**, Illustration of PSB using the energy levels in the quadruple quantum dot plaquette for DQD 2-6 (**i**) and 3-7 (**j**), respectively. The hole numbers are indicated as $$\left(\begin{array}{rc}{n}_{1},&{n}_{2}\\ {n}_{5},&{n}_{6}\end{array}\right)$$ for **i** and $$\left(\begin{array}{rc}{n}_{3},&{n}_{4}\\ {n}_{7},&{n}_{8}\end{array}\right)$$ for **j**, and the subscripts *S* and *T* show the two-spin states of holes in the quantum dots indicated by bold numbers, respectively. The solid arrows show fast spin-conserving tunnelling while the dashed arrows show suppressed tunnelling due to PSB. Here we take pair 2-6 as an example to explain the readout process of the inner spin pairs. First, we align DQD 1-5 at the charge stability boundary between (2,0) and (2,1), as shown by the white dot in **e**, and then pulse *ε*_26_ from negative to positive. We subsequently find a shaded region between (0,1) and (0,2) in the diagram for DQD 2-6, which is caused by PSB in DQD 5-6. The mechanism is shown in **i**: when we pulse DQD 2-6 to the point where *S*(0, 2) is lower in energy than (0, 1), the holes in DQD 2-6 moves across to DQD 5-6, irrespective of the spin states. Subsequently, the conventional PSB mechanism in DQD 5-6 allows *S*(1, 1) to transition to *S*(0, 2), while the triplets *T*(1, 1) have to remain in the (1,1) charge state. In this way, we indirectly realize spin-to-charge conversion for the two spins initially in DQD 2-6. Actually, *S*(1, 1) in DQD 2-6 can also directly tunnel to *S*(0, 2) inside the same DQD, as seen by the curved arrow in **i**. The mechanism to measure DQD 3-7 is analogous.

As shown in Fig. 1a,b, the 2 × 4 quantum dot ladder is fabricated in a germanium quantum-well heterostructure^45^. The gate pattern and substrate have the same design as that in ref. ^36^ (Methods). Linear combinations of plunger gate voltages {*P*_*i*_} allow us to set the overall electrochemical potential of each DQD *μ*_*ij*_ = (v*P*_*i*_ + v*P*_*j*_)/2 and the interdot detuning *ε*_*ij*_ = (v*P*_*i*_ − v*P*_*j*_)/2. The prefix ‘v’ indicates that the physical gate voltages are virtualized to compensate the crosstalk on the dot potentials^10^ (see Supplementary Note II for the virtual gate matrix). Single-hole occupation of each quantum dot in the array is confirmed by measuring the charge stability diagrams using sensors S_BL_ and S_BR_ (Extended Data Fig. 1).

## Singlet–triplet qubit and energy spectroscopy

We encode the qubit into the two-spin singlet-triplet states, $\left\vert {\mathrm{S}}\right\rangle$ and $\left\vert {\mathrm{T}}_{-}\right\rangle$, of the DQDs along the rungs of the quantum dot ladder, with the singlet $\left\vert {\mathrm{S}}\right\rangle =(\left\vert \uparrow \downarrow \right\rangle -\left\vert \downarrow \uparrow \right\rangle )/\sqrt{2}$ and the lowest-energy triplet $\left\vert {\mathrm{T}}_{-}\right\rangle =\left\vert \downarrow \downarrow \right\rangle$. Thus Q1, Q2, Q3 and Q4 are formed using DQDs 1–5, 2–6, 3–7 and 4–8, respectively. Qubit readout is achieved by pulsing the corresponding DQD to the PSB regime, that is in the (0,2) or (2,0) regime but close to (1,1). This regime converts the singlet and triplet states into distinct charge states, which are then measured through the charge sensor (see Extended Data Fig. 1 for details). The single-qubit Hamiltonian can be written as

1 $${H}_{\mathrm{ST}_{-}}=\frac{{\overline{E}}_{z}-J}{2}{\sigma }_{z}+\frac{{\Delta }_{\mathrm{ST}_{-}}}{2}{\sigma }_{x},$$

where *σ*_*x*_ and *σ*_*z*_ are the Pauli matrices, *J* = *J*(*ε*_*ij*_,v*b*_*ij*_) is the exchange coupling between two spins, which is a function of both the detuning *ε*_*ij*_ and the barrier gate voltage v*b*_*ij*_, and ${\overline{E}}_{z}={\overline{g}}_{ij}{\mu }_{\mathrm{B}}B$ is the average Zeeman energy of the two hole spins in the DQD, with ${\overline{g}}_{ij}$ the average *g*-factor, *μ*_B_ the Bohr magneton, and *B* the magnetic field strength. Unless indicated otherwise, an in-plane magnetic field (up to alignment precision) of *B* = 5 or 10 mT is applied to the device. The intrinsic spin–orbit interaction for holes in germanium couples the states $\left\vert {\mathrm{S}}\right\rangle$ and $\left\vert {\mathrm{T}}_{-}\right\rangle$ with an energy ${\Delta }_{\mathrm{ST}_{-}}$.

Figure 1c–e shows the energy levels of the two-spin $\left\vert S\right\rangle$ and $\left\vert {T}_{-}\right\rangle$ states in a DQD with $J({\varepsilon }_{ij}=0) < {\overline{E}}_{z},\,J({\varepsilon }_{ij}=0)={\overline{E}}_{z},\,J({\varepsilon }_{ij}=0) > {\overline{E}}_{z}$, respectively. The other two-spin states are $\left\vert {\mathrm{T}}_{0}\right\rangle =(\left\vert \uparrow \downarrow \right\rangle +\left\vert \downarrow \uparrow \right\rangle )/\sqrt{2}$ and $\left\vert {\mathrm{T}}_{+}\right\rangle =\left\vert \uparrow \uparrow \right\rangle$. In a DQD, we use (*n*_L_,*n*_R_) to denote the charge number in the left (*n*_L_) and right (*n*_R_) dot. By adjusting the detuning *ε*_*ij*_ of the DQD from negative to positive, we can change the charge state from (2,0) to (1,1) and then to (0,2), as indicated by the labels on top of each diagram, and the energy levels of the two-spin states in the DQD will change accordingly. As shown in Fig. 1c, when *J*(*ε*_*ij*_ = 0) is smaller than ${\overline{E}}_{z}$, the singlet $\left\vert {\mathrm{S}}\right\rangle$ crosses the triplet $\left\vert {\mathrm{T}}_{-}\right\rangle$ twice in the (1,1) regime. Due to intrinsic spin–orbit coupling, these are in fact avoided crossings with a gap ${\Delta }_{\mathrm{ST}_{-}}$, where the states $\left\vert {\mathrm{S}}\right\rangle$ and $\left\vert {\mathrm{T}}_{-}\right\rangle$ are admixed. As *J*(*ε*_*ij*_ = 0) increases, the two anticrossings approach each other and eventually merge into one, as shown in Fig. 1d. When *J*(*ε*_*ij*_ = 0) increases even further (Fig. 1e), $\left\vert {\mathrm{S}}\right\rangle$ and $\left\vert {\mathrm{T}}_{-}\right\rangle$ no longer exhibit an avoided crossing.

Experimentally, we probe the position of the avoided crossings as a function of the barrier gate voltage v*b*_*ij*_ that controls *J* for each qubit. This results in the parabola-like patterns, also called spin mixing maps^19,46^, in Fig. 1f–i. As expected, when v*b*_*ij*_ is tuned from positive to negative, *J* increases and the positions of the S–T_−_ anticrossings move inwards before disappearing. The asymmetry visible in these panels can arise from imperfect virtualization of the barrier gates or from a detuning-dependent Zeeman energy^47^ (Supplementary Note III).

## Universal single-qubit control

**Fig. 2:**
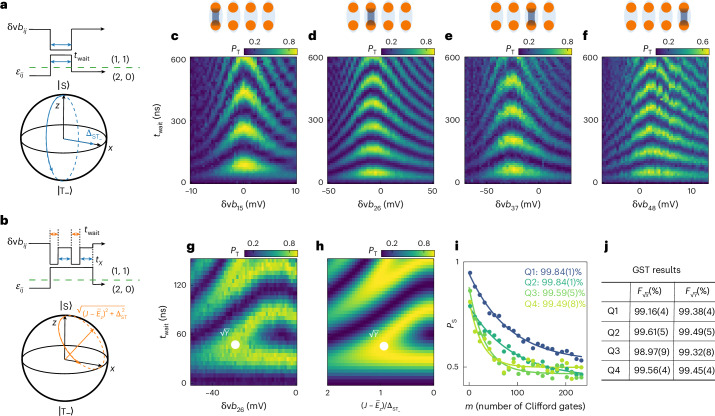
Universal single-qubit control of four singlet–triplet qubits. **a**,**b**, The pulse schemes used for *x*-axis control (**a**) and *y*-axis control (**b**). In the experiments, the detuning pulse in **a** and **b** has a 20 ns ramp (not shown) from (2,0) to (1,1), similar to the pulse used for the energy spectroscopy. **c**–**f**, Experimental results for *x*-axis rotations of Q1 (**c**), Q2 (**d**), Q3 (**e**) and Q4 (**f**), showing measured triplet probabilities *P*_T_ as a function of *t*_wait_ and the corresponding barrier voltage *δ*v*b*_*ij*_. **g**, Measured *P*_T_ for the sequence shown in **b** as a function of *t*_wait_ and the barrier voltage change *δ*v*b*_26_. The position where $\sqrt{Y}$ is properly calibrated is indicated by a white dot. **h**, The numerically computed *P*_T_ as a function of *t*_wait_ and the ratio of the *z*-axis component to the *x*-axis component, $(J-{\overline{E}}_{z})/{\Delta }_{\mathrm{ST}_{-}}$. The parameters used for the calculation are extracted from **g**. **i**, Single-qubit RB data for Q1–Q4, with *P*_S_ the measured probabilities of the target state. The 95% confidence intervals based on statistical fluctuations are smaller than the data points. The numbers in the legend are the extracted average gate fidelities, which are obtained from the Clifford gate fidelities using a ratio of 3.625 (see Extended Data Table 1 for the detailed gate decomposition). The errors represent the 68% confidence intervals (Methods). **j**, Table showing the single-qubit gate fidelities of Q1–Q4 measured by GST. The errors represent the 95% confidence intervals computed using the Hessian of the log-likelihood function^51^. All the data above are measured at *B* = 5 mT.

**Extended Data Fig. 2:**
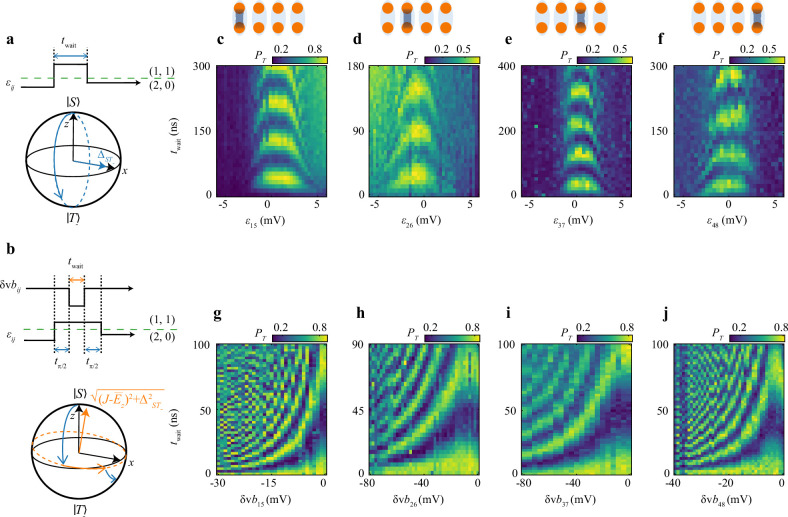
Data of two-axis qubit control around the *x*- and *z*-axis, measured at *B* = 10 mT. **a**,**b**, Pulse scheme and Bloch sphere illustration of *x*-axis and *z*-axis evolution of *S* − *T*_−_ qubits. The straight blue and orange arrows show the corresponding rotation axis. The *x*-axis rotations are set by the *S* − *T*_−_ coupling, ${\Delta }_{S{T}_{-}}$. For large *J* such that $J-{\overline{E}}_{z}\gg {\Delta }_{S{T}_{-}}$, the rotation axis tilts towards the *z*-axis. The rotation is never exactly around the *z*-axis due to the presence of a finite ${\Delta }_{S{T}_{-}}$, yet, sufficiently orthogonal control is possible when $(\,J-{\overline{E}}_{z})\gg {\Delta }_{S{T}_{-}}$. In **b**, we illustrate a Ramsey-like pulse sequence used to demonstrate *z*-axis control. We first initialize the qubit into a singlet, perform a *π*/2 rotation around the *x*-axis of duration *t*_*π*/2_, and then change *J* diabatically by pulsing the corresponding barrier gate by an amount *δ*v*b*_*ij*_ to implement a *z*-axis rotation. Finally, we perform another *π*/2 operation around the *x*-axis and project the qubit into the *S* − *T*_−_ basis for spin readout. **c-f**, Experimental results for *x*-axis rotations of each qubit, showing measured triplet probabilities *P*_*T*_ as a function of *t*_wait_ and the detuning voltage *ε*_*ij*_. **g-j**, Experimental results for *z*-axis rotations of each qubit, showing *P*_*T*_ as a function of *t*_wait_ and the barrier voltage change *δ*v*b*_*ij*_. The oscillation frequency is given by ${f}_{S{T}_{-}}=\sqrt{{(J-{\overline{E}}_{z})}^{2}+{\Delta }_{S{T}_{-}}^{2}}/h$, where *h* is Planck’s constant. We note that the outer two barrier gates v*b*_15_ and v*b*_48_ have a stronger effect on the corresponding *J*_*ij*_ than the inner barrier gates v*b*_26_ and v*b*_37_. This may be explained by additional residual resist below the inner barrier gates, which are fabricated in the last step^36^, and by the different fan-out routing for the outer barrier gates (see Fig. 1a,b in the main text). Within the tuning range of the barrier gate, the highest ratio $(\,J-{\overline{E}}_{z})/{\Delta }_{S{T}_{-}}$ amounts to around 20 for the outer qubits Q1 and Q4 and about 10 for the inner qubits Q2 and Q3 (see Supplementary Information IV for more details).

**Extended Data Fig. 3:**
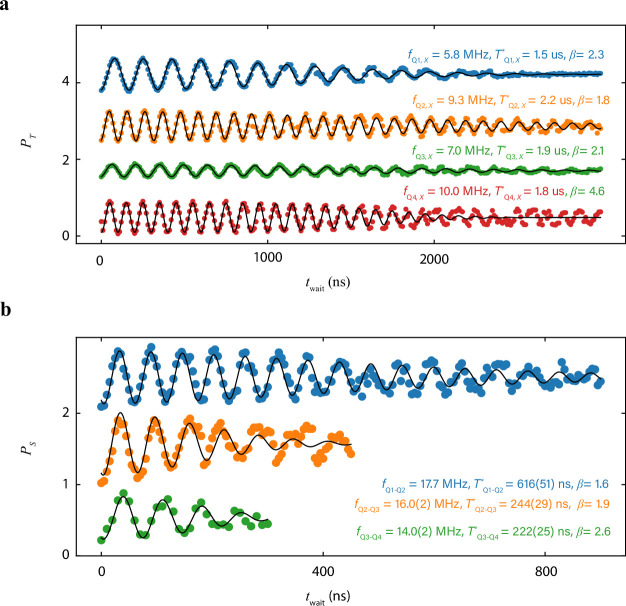
Decoherence times of the qubits under control. **a**, Measured triplet probabilities *P*_*T*_ of long-time evolutions around the *x*-axis for Q1–Q4 at *B*=5 mT. **b**, Measured singlet probabilities *P*_*S*_ as a function of the evolution time *t*_wait_ at the centre of the chevron patterns of the SWAP oscillations for each pair of qubits at *B*=5 mT. The data of **a** and **b** are fitted with a function of the form ${P}_{T}={P}_{0}+A\cos (2\pi ft+\phi )\exp [-{(t/{T}^{* })}^{\beta }]$, where *P*_0_, *A*, *β*, *f*, T^*^ are fitting parameters. Here *f* refers to the oscillation frequency, T^*^ refers to ${T}_{x}^{* }$, the coherence time under *x*-axis rotations, or ${T}_{Qi-Qj}^{* }$, the coherence time under SWAP oscillations between adjacent qubits. Furthermore, the parameter *β* determines the shape of the decay envelope, and the fitted values are shown in the inset. *β* provides insight into the noise spectrum within the system: when quasi-static or low-frequency noise dominates, *β* = 2, leading to Gaussian decay; whereas when high-frequency noise prevails, *β* = 1, resulting in exponential decay. The extracted *β* for the *x*-axis rotations of Q1-Q3 and all the SWAP oscillations are close to 2, indicating the dominance of low-frequency noise in this system. Notice the large value of *β* for the *x*-axis rotations of Q4 may result from the fitting error. The errors represent the 68% confidence intervals obtained from fitting.

**Extended Data Fig. 4:**
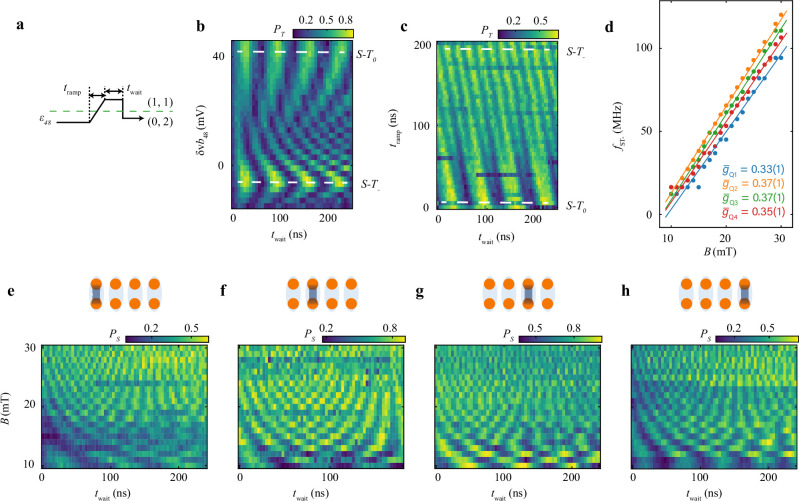
Coherent control of singlet-triplet states under different conditions and the average *g*-factor. **a**, Pulse scheme to measure *S* − *T*_−_ and *S* − *T*_0_ oscillations. A square pulse is used with a ramp-in time *t*_ramp_ and waiting time *t*_wait_. **b**, Measured triplet probabilities of DQD 4-8 as a function of *t*_wait_ and the barrier voltage amplitude *δ*v*b*_48_ with *t*_ramp_ = 20 ns. As mentioned in the main text, a block pulse along the detuning axis with 20-nanosecond ramp time (*t*_ramp_) from (0,2) to (1,1) is adiabatic with respect to the tunnel coupling but diabatic with respect to the *S* − *T*_−_ anticrossing. Therefore, we can drive *x*-rotations of *S* − *T*_−_ qubits when the pulse amplitude reaches zero detuning with $J({\varepsilon }_{ij}=0) \sim \overline{{E}_{z}}$ (see Fig. 1d in the main text). However, when we increase the barrier voltage change *δ*v*b*_48_ until $J({\varepsilon }_{ij}=0) < \overline{{E}_{z}}$ (*δ*v*b*_48_ ~ 40 mV), the *S* − *T*_−_ anticrossings appear away from zero detuning (see Fig. 1c in the main text), thus the same pulse does not produce *x*-axis oscillations of the *S* − *T*_−_ qubit. Moreover, under this condition, the *S* − *T*_0_ splitting is reduced and the 20-nanosecond ramp time eventually becomes diabatic with respect to the *S* − *T*_0_ splitting. As a result, the singlet state will rotate between the *S* and *T*_0_ states under the Zeeman energy difference between the two dots *ΔE*_*z*_. **c**, Measured triplet probabilities of DQD 4-8 as a function of *t*_wait_ and *t*_ramp_ with *δ*v*b*_48_ = 40 mV. When *t*_ramp_ is small, the observed oscillations are between *S* and *T*_0_; however, when *t*_ramp_ is increased until the pulse is adiabatic with respect to the *S* − *T*_0_ splitting (over 100 ns), the *S* − *T*_0_ oscillations can no longer be observed. Such a long ramp time can rotate the initial state to a superposition state between *S* and *T*_−_ states, and *z*-axis rotations of the *S* − *T*_−_ qubit become visible^41^. Therefore, we also observe a transition of *S* − *T*_0_ oscillations and *S* − *T*_−_ oscillations as a function of *t*_ramp_ in the figure. **d**, The rotation frequency ${f}_{S{T}_{-}}$ of each qubit as a function of the magnetic field strength *B*. When the external magnetic field strength is varied while keeping the gate voltages fixed, the frequency of these *S* − *T*_−_ oscillations increases nearly linearly with the field due to the contribution from Zeeman energy in ${f}_{S{T}_{-}}$. From the slope, we extract ${\overline{g}}_{ij}$ for the four qubits as shown in the inset. The errors represent the 68% confidence intervals obtained from fitting. The data are acquired using the fast Fourier transform (FFT) of time-domain oscillations in **e-h**. **e-h**, Measured singlet probabilities *P*_*S*_ of Q1 (**i**), Q2 (**j**), Q3 (**k**), and Q4 (**l**) as a function of *t*_wait_ and magnetic field strength *B*. The rotations are induced using the pulse scheme of panel **a** with *t*_ramp_ = 100 ns.

**Extended Data Fig. 5:**
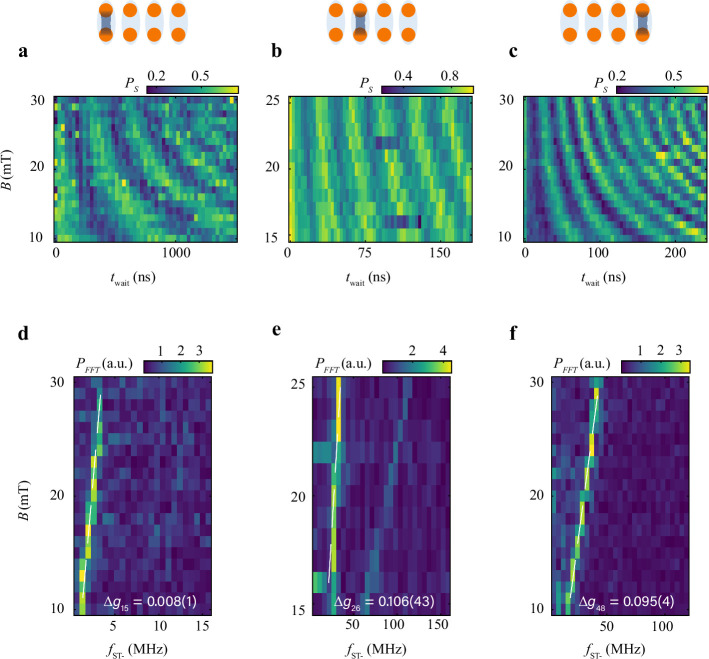
Coherent control of *S* − *T*_0_ states and *g*-factor differences. **a-c**, Measured singlet probabilities *P*_*S*_ as a function of *t*_wait_ and the magnetic field strength *B* for Q1 (**c**), Q2 (**d**), and Q4 (**e**) during *S* − *T*_0_ oscillations. Here *t*_ramp_ is set to zero and the barrier gate voltage is set such that $J({\varepsilon }_{ij}=0) < \overline{{E}_{z}}$ to suppress unwanted *S* − *T*_−_ oscillations. **d-f**, The fast Fourier transforms of the data in **a-c**, with a signal that can be line-fitted using the *g*-factor difference *Δg*_*ij*_ (inset) of two dots. The errors represent the 68% confidence intervals obtained from fitting. For Q3, we didn’t find *S* − *T*_0_ oscillations, which may be because the corresponding *Δg* is too low to detect.

With the knowledge of the energy spectrum of the four S–T_−_ qubits, we next implement *x*- and *y*-axis control of each qubit, which is necessary and sufficient for universal single-qubit control (for *z*-axis control, see Extended Data Fig. 2). By operating the qubit in the regime where $J={\overline{E}}_{z}$, the first term of equation (1) goes to zero and ${\Delta }_{\mathrm{ST}_{-}}$ rotates the qubit around the *x* axis in the Bloch sphere (Fig. 2a). Furthermore, we tune the barrier voltage to obtain $J={\overline{E}}_{z}$ at zero detuning, which is a symmetry point where the effect of detuning noise is strongly suppressed^48,49^. The pulse scheme for testing *x*-axis control is shown in Fig. 2a: first we initialize the qubit into a singlet by starting in the (2,0) (or (0,2)) regime, then pulse the detuning to the centre of the (1,1) regime where $J({\varepsilon }_{ij}=0)={\overline{E}}_{z}$, next allow the qubit to evolve for a variable time *t*_wait_, and finally pulse the detuning back to a point in the (2,0) (or (0,2)) regime for spin readout via PSB. The measured rotations of Q1–Q4 as a function of the corresponding barrier gate voltage are shown in Fig. 2c–f. By choosing the point where the oscillation speed is the slowest, that is, at the S–T_−_ anticrossing, the qubits rotate around the *x* axis. Long-timescale *x* rotations are shown in Extended Data Fig. 3a. At *B* = 5 mT, the dephasing times are in the range of 1.5–2.2 μs for Q1–Q4, mostly limited by low-frequency or quasi-static noise (see Supplementary Note IV for additional data taken at *B* = 10 mT). The average *g*-factors were estimated by scanning the magnetic field, yielding ${\overline{g}}_{ij}$ in the range of 0.33(1)–0.37(1). For more experimental details and information on the *g*-factors, see Extended Data Figs. 4 and 5.

To realize *y*-axis control, we use the relationship $\sqrt{Y}=XH$, where $\sqrt{Y}$ stands for a π/2 rotation around the *y* axis, *X* stands for a π rotation around the *x* axis, and *H* refers to the Hadamard gate. For the Hadamard gate, we increase *J* to a value where $(\,J-{\overline{E}}_{z})={\Delta }_{\mathrm{ST}_{-}}$, causing the qubit to rotate around an axis halfway between the *x* and *z* axes. A rotation angle of π corresponds to the implementation of the Hadamard gate. To calibrate the rotation axis and rotation angle of the Hadamard gate, we concatenate two $\sqrt{Y}$ gates and evaluate the probability of having flipped the qubit (Fig. 2b). Specifically, we first initialize the qubit into a singlet, then change *J* diabatically by pulsing the corresponding barrier gate by an amount *δ*v*b*_*ij*_ for a time *t*_wait_ to implement the Hadamard gate, and finally apply an *X* gate for a time *t*_*X*_ (aiming to stay at the detuning symmetry point throughout^48,49^). This combination is repeated such that a *Y* gate is expected for the right choice of *δ*v*b*_*ij*_ and *t*_wait_, the two parameters that control the Hadamard gate. This procedure is illustrated for Q2 in Fig. 2g. The white dot shows the position where the sequence of Fig. 2b produces a *Y* gate. Running only the first half of the sequence implements a $\sqrt{Y}$ gate. A corresponding numerical simulation result, displayed in Fig. 2h, shows a similar pattern as the experimental result.

**Extended Data Fig. 6:**
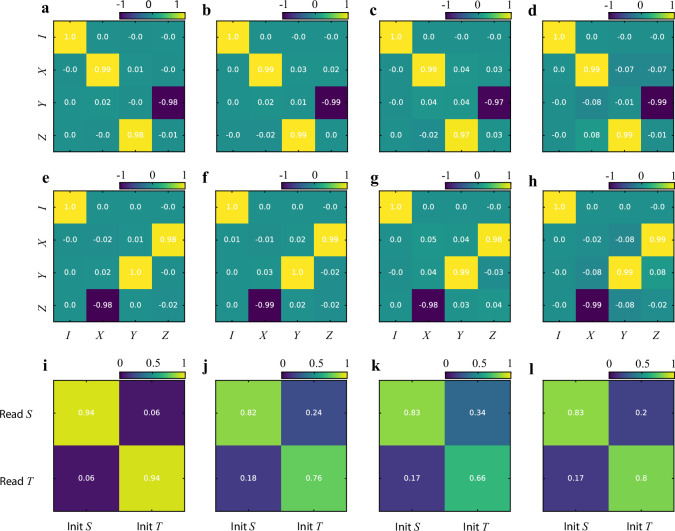
Results of single-qubit gate set tomography. **a-h** Single-qubit Pauli transfer matrices (PTM) of the $\sqrt{X}$ gate (**a-d**) and $\sqrt{Y}$ gate (**e-h**) for Q1–Q4 (from left to right) obtained from gate set tomography. **i-l**, Estimated state preparation and measurement (SPAM) error probabilities from GST results for Q1–Q4, using the same method as used in^52^. We find that the SPAM errors of Q2 and Q3 are worse than those of Q1 and Q4. There are two reasons. Firstly, the indirect PSB mechanism is more sensitive to the readout point we chose and the idling point of the other qubit. In particular, the readout fidelity of the triplet is lower when triplet relaxation is faster at the readout point. Secondly, the slower tunnelling rate from (0,2) or (2,0) to (1,1) causes an initialization error in some cases. Also, the instability of the charge sensor can contribute to the readout error, which makes the readout visibility vary between different measurements.

Using the $\sqrt{X}$ and $\sqrt{Y}$ gates, we perform randomized benchmarking (RB) to obtain the average gate fidelities (Fig. 2i). All four qubits yield average gate fidelities at or above 99%, extracted from the Clifford gate fidelities^50^. We also measure the $\sqrt{X}$ and $\sqrt{Y}$ gate fidelities with gate set tomography (GST)^51,52^, the results of which are summarized in Fig. 2j. Overall, most of the fidelities in GST results are slightly lower than those from RB. These fidelity differences may stem from the presence of low-frequency noise in our system, which causes different uncertainties in the fidelity estimates between GST and RB^53^. Full details on the quantum process for those two gates, derived from GST, are given in Extended Data Fig. 6 and Supplementary Note VIII.

## Two-qubit gate

To realize universal control of the full four-qubit register, we need to complement single-qubit gates with two-qubit entangling gates. Assuming isotropic exchange interactions between adjacent S–T_−_ qubits, the two-qubit Hamiltonian in the basis of $\{\left\vert {\mathrm{SS}}\right\rangle ,\,\left\vert {\mathrm{ST}}_{-}\right\rangle ,\,\left\vert {\mathrm{T}}_{-}{\mathrm{S}}\right\rangle \,{\rm{and}}\,\left\vert {\mathrm{T}}_{-}{\mathrm{T}}_{-}\right\rangle \}$ can be written as:

2 $$\begin{array}{ll}{H}_{{\rm{2Q}}}\,=\,\displaystyle\frac{({\overline{E}}_{z,ij}\,-\,{J}_{ij}){\sigma }_{z}^{ij}\,+\,{\Delta }_{{\mathrm{ST}}_{-},ij}{\sigma }_{x}^{ij}}{2}\\\qquad\quad+\,\displaystyle\frac{({\overline{E}}_{z,kl}\,-\,{J}_{kl}){\sigma }_{z}^{kl}\,+\,{\Delta }_{{\mathrm{ST}}_{-},kl}{\sigma }_{x}^{kl}}{2}\\\qquad\quad+\displaystyle\,\frac{{J}_{{\rm{coup}}}}{4}[{\sigma }_{x}^{ij}{\sigma }_{x}^{kl}+{\sigma }_{y}^{ij}{\sigma }_{y}^{kl}+\frac{1}{2}({\sigma }_{z}^{ij}-I)({\sigma }_{z}^{kl}-I)],\end{array}$$

where *ij* and *kl* refer to the respective qubit dot pair, and the interqubit coupling *J*_coup_ = (*J*_*ik*_ + *J*_*jl*_)/2. The coupling term is reminiscent of two well-known interaction Hamiltonians. If the factor 1/2 of the *σ*_*z*_*σ*_*z*_ coupling term were 1 instead, we recover the exchange Hamiltonian that generates the SWAP gate and the universal $\sqrt{{\rm{SWAP}}}$ gate. If that factor were zero, only the flip-flop terms would survive, which generate the iSWAP and $\sqrt{{\rm{iSWAP}}}$ gate. The coupling Hamiltonian in equation (2) thus generates a SWAP-style gate that is not a standard two-qubit gate but is also universal from the perspective of quantum computing (Supplementary Note VII). For simplicity, we call it a SWAP gate in the remainder of this work.

**Fig. 3:**
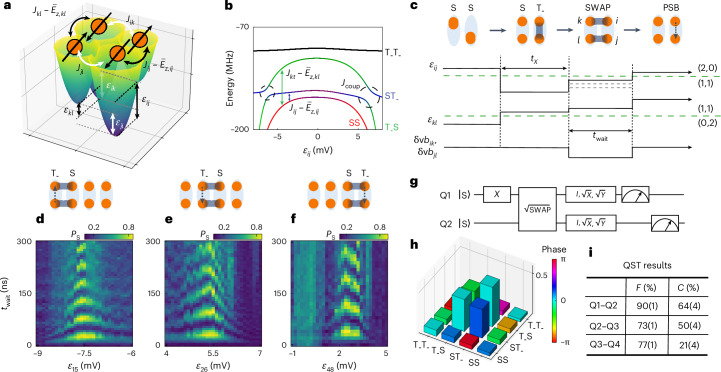
Two-qubit interactions across the quantum dot ladder. **a**, A representation of two connected DQDs. The S–T_−_ qubits have a splitting of ${J}_{ij}-{\overline{E}}_{z,ij}$ and ${J}_{kl}-{\overline{E}}_{z,kl}$ (neglecting ${\Delta }_{{\mathrm{ST}}_{-}}$), which are controlled by the detunings *ε*_*ij*_ and *ε*_*kl*_, respectively. The qubit–qubit coupling *J*_coup_ is an average of *J*_*ik*_ and *J*_*jl*_ between the corresponding dots, which are controlled by *ε*_*ik*_ and *ε*_*jl*_. **b**, The energy levels of two-qubit states, where we fix *ε*_*kl*_ to be positively large and scan *ε*_*ij*_. At the positions where ${J}_{ij}-{\overline{E}}_{z,ij}$ equals ${J}_{kl}-{\overline{E}}_{z,kl}$, an anticrossing with a gap *J*_coup_ forms (black dashed circles), which can be used to induce SWAP oscillations between $\left\vert {\mathrm{ST}}_{-}\right\rangle$ and $\left\vert {\mathrm{T}}_{-}{\mathrm{S}}\right\rangle$. The parameters used in this calculation are based on the experimental results for Q3–Q4 shown in Supplementary Fig. 4c. **c**, The pulse scheme for SWAP operations. We start in (0,2) or (2,0), at large positive or negative detuning, and diabatically pulse one qubit to (1,1) at modest detuning such that it remains in $\left\vert {\mathrm{S}}\right\rangle$, and pulse the other qubit to zero detuning where a π rotation for a time *t*_*X*_ takes it to $\left\vert {\mathrm{T}}_{-}\right\rangle$ (other qubits are either initialized to singlets by pulsing back and forth to (0,2) or (2,0), or remain in the (1,1) regime all the time). At this point, the qubits are set to $\left\vert {\mathrm{ST}}_{-}\right\rangle$ or $\left\vert {\mathrm{T}}_{-}{\mathrm{S}}\right\rangle$. Next, we pulse the detunings of both qubits to make their energies resonant, while at the same time activating *J*_*ik*_ and *J*_*jl*_. This will kickstart SWAP oscillations between the two qubits. The dashed lines in the pulse of *ε*_*ij*_ show that we scan the detuning of one qubit to find the condition for SWAP operations. After an evolution time *t*_wait_, we pulse the detunings to the PSB readout configuration for one of the qubits. **d**–**f**, The experimental results of SWAP oscillations, showing measured singlet probabilities *P*_S_ as a function of operation time *t*_wait_ and the detuning voltage *ε*_*ij*_ for Q1–Q2 (**d**), Q2–Q3 (**e**) and Q3–Q4 (**f**). The initial states of two qubits (before the SWAP oscillations) are denoted on the top, and the qubit pair that is read out is indicated by the dashed arrow showing the readout pulse direction. **g**, The quantum circuit used to create a generalized Bell state between Q1 and Q2 and to characterize it via QST. **h**, Measured two-qubit density matrix of Q1–Q2, after removal of SPAM errors and using maximum-likelihood estimation (MLE). **i**, State fidelities and concurrence estimated from the density matrices of the Bell states of Q1–Q2, Q2–Q3 and Q3–Q4. The errors show the uncertainty with 68% confidence intervals (Methods). The data in panels **d**–**f** and **h**,**i** are measured at *B* = 5 mT (see Supplementary Note V for additional data taken at *B* = 10 mT).

To activate the SWAP gate, we equalize the energy splittings of two qubits and turn on *J*_*ik*_ and *J*_*jl*_ such that the flip-flop terms can exchange the qubit populations (note that if the qubit energies were set very different from each other, a controlled-Z or CZ gate would result instead). Our strategy for meeting both requirements at the same time is to use the interdot detuning of both qubits^12,13^. A typical potential landscape for the two qubits in DQD *ij* and *kl* is shown in Fig. 3a, where we pulse *ε*_*ij*_ to large positive and *ε*_*kl*_ to large negative detuning. The detunings *ε*_*ik*_ and *ε*_*jl*_, which control the interactions between the qubits, are then automatically increased as well. Therefore, all the exchange interactions involved are enhanced simultaneously and the effect of the single-qubit terms *σ*_*x*_ is made negligible. In practice, we fix the (large) detuning of one qubit and finetune that of the other to find the position where two qubits have equal energy splittings. This is illustrated by the energy spectrum in Fig. 3b, where we fix the detuning *ε*_*kl*_ to a large negative value and scan the detuning *ε*_*ij*_. We see that the states $\left\vert {\mathrm{ST}}_{-}\right\rangle$ and $\left\vert {\mathrm{T}}_{-}{\mathrm{S}}\right\rangle$ anticross at the two positions where ${J}_{ij}-{\overline{E}}_{z,ij}$ is equal to ${J}_{kl}-{\overline{E}}_{z,kl}$. The gap size is given by *J*_coup_. Since *ε*_*ik*_ and *ε*_*jl*_, which control *J*_coup_ via *J*_*ik*_ and *J*_*jl*_, are also dependent on *ε*_*ij*_, the sizes of the two gaps are not necessarily the same.

**Extended Data Fig. 7:**
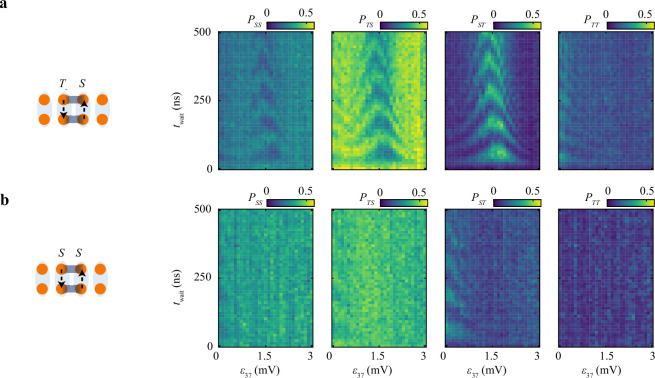
Sequential readout and joint probabilities of two qubits under SWAP oscillations. **a**,**b**, Sequentially measured probabilities *P*_*SS*_, *P*_*TS*_, *P*_*ST*_ and *P*_*TT*_ of Q2 and Q3 as a function of *t*_wait_ and the detuning of Q3, *ε*_37_, after initializing Q2–Q3 into $\left\vert {T}_{-}S\right\rangle$ (**a**) and $\left\vert SS\right\rangle$ (**b**). The data is acquired at *B* = 5 mT. In **a**, the out-of-phase signals in *P*_*TS*_ and *P*_*ST*_ observed around *ε*_37_ = 1.5 mV are the result of the SWAP oscillations between these two qubits. A similar signal to *P*_*TS*_ but with lower visibility is observed in *P*_*SS*_, which can be explained by the higher triplet readout error for Q2 than for Q3. The sequential readout is achieved by pulsing the barrier gate *δ*v*b*_26_ to -60 mV, where we measure Q3 first for a duration of 20 *μ*s and simultaneously keep Q2 in the centre of (1,1) with sufficiently large *J*, where the Hamiltonian eigenbasis corresponds to the qubit readout basis^12,13,30^. In the next step of the sequence, Q2 is measured. In panel **b**, we do not observe any apparent leakage to $\left\vert TT\right\rangle$ but only see signs of single-qubit rotations at low detunings. This is expected given that, in this regime, the states $\left\vert TT\right\rangle$ are far away in energy from the other states (see Fig. 3b in the main text).

Figure 3c shows an example of the pulse scheme we use in the experiment to observe two-qubit SWAP oscillations. Figure 3d–f shows the resulting SWAP oscillations for Q1–Q2, Q2–Q3 and Q3–Q4. Chevron-style patterns are observed with the energies of the two qubits aligned in the middle of the patterns. At the centre of the chevron patterns, we measured the dephasing times of 222(25)–616(51) ns (Extended Data Fig. 3b). Looking closely, the chevron patterns are not symmetric. This can be understood by the fact that the qubit energy does not vary linearly with detuning. In some panels, single-qubit oscillations around an axis close to the *x* axis are also observed, such as the data at *ε*_26_ = 4 mV in Fig. 3e. These *ε*_*ij*_ values are close to zero interdot detuning, and when *J*(*ε*_*ij*_ = 0) is not much larger than ${\overline{E}}_{z}$, such rotations are expected. Data based on simultaneous readout of two qubits under SWAP oscillations can be found in Extended Data Fig. 7. We note that SWAP oscillations between $\left\vert {\mathrm{ST}}_{-}\right\rangle$ and $\left\vert {\mathrm{T}}_{-}{\mathrm{S}}\right\rangle$ were also observed in previous research on simulating the dynamics of an antiferromagnetic spin chain and resonating-valence-bond states based on the Heisenberg model in four-quantum-dot systems^12,13^.

**Extended Data Fig. 8:**
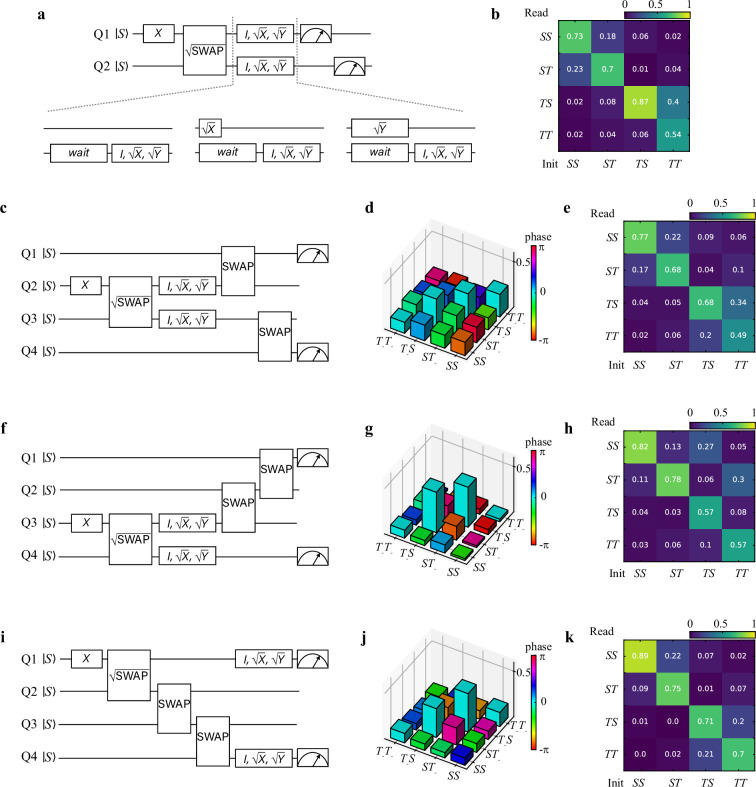
Quantum gate circuit and results for quantum state tomography of the Bell states. **a**,**c**,**f**,**i**, Quantum circuit used to prepare and characterize a Bell state for different qubit pairs. In **a**, we plot the details of the single-qubit basis changes after the generation of the Bell state, where we apply a fixed wait time before performing gates on Q2 to keep its phase consistent through all the experiments. In **c**, SWAP gates are used to transfer the state of Q2 to Q1 and that of Q3 to Q4. Next Q1 and Q4 are measured simultaneously using two sensors. In **f**, two consecutive SWAP gates are used to transfer Q3 to Q1. Next Q1 and Q4 are measured simultaneously using two sensors as well. In **i**, the quantum information is transferred from Q2 to Q4 before performing the single-qubit gates for basis changes. This allows us to quantify the entanglement between Q1 and Q4 after state transfer. **d**,**g**,**j**, Two-qubit density matrices obtained from the corresponding quantum circuit after removal of measurement errors and using MLE for Q2–Q3 (**d**), Q3–Q4 (**g**) and Q1–Q4 (**j**) (Fig. 3h shows the density matrix for Q1–Q2). Measurement errors were removed based on the SPAM matrices. These matrices include not only measurement errors but also initialization errors, hence we are overcorrecting. The fact that initialization errors for most qubits were much smaller than measurement errors combined with the fact that MLE forces the resulting density matrix to be physical, helps ensure a reliable outcome. If we don’t attempt to remove readout errors, the density matrices show state fidelities and concurrences of 71.3(6)% and 9(2)% for Q1–Q2, 64.2(6)% and 10(2)% for Q2–Q3, 64.6(7)% and 0(0)% for Q3–Q4 and 64.4(9)% and 0(0)% for Q1–Q4. The errors represent the 68% confidence intervals (Methods). **b**,**e**,**h**,**k**, SPAM matrices used in the quantum state tomography analysis of Q1–Q2 (**b**), Q2–Q3 (**e**), Q3–Q4 (**h**) and Q1–Q4 (**k**). The SPAM matrices of Q1–Q2 and Q1–Q4 were measured directly by initializing them to the indicated states and measuring the corresponding pair in a single-shot manner. For Q2–Q3 or Q3–Q4, we initialized the qubits to the indicated states and measured the state of Q1–Q4 after the SWAP operations. These SPAM matrices do include errors from the SWAP operations.

**Extended Data Fig. 9:**
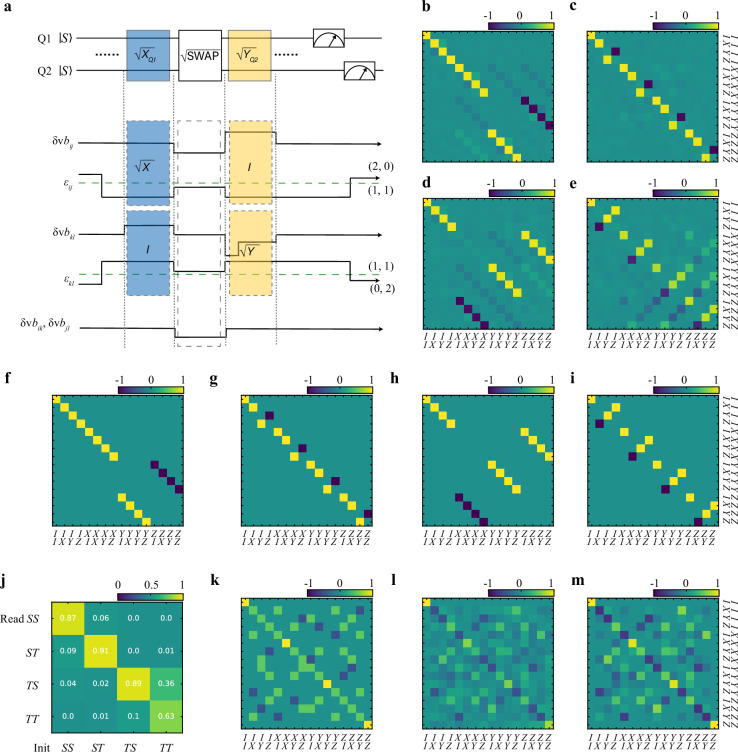
Measurement sequence and results of two-qubit gate set tomography. **a**, Illustration of the gate voltage pulses for a two-qubit circuit. Performing a single-qubit gate in the two-qubit space is nontrivial since during the time one qubit is undergoing an operation, the idling qubit could suffer from unwanted rotations and crosstalk. To solve this problem, we pulse the idle qubit to an operating point where it completes a 2*π* rotation during the time needed to operate on the other qubit (see Supplementary Information VIII for more details). **b-e**, Measured PTMs obtained from GST for single-qubit gates in the two-qubit space, including ${\sqrt{X}}_{Q1}$ (**b**), ${\sqrt{X}}_{Q2}$ (**c**), ${\sqrt{Y}}_{Q1}$ (**d**) and ${\sqrt{Y}}_{Q2}$ (**e**). **f-i**, The ideal PTMs from GST for single-qubit gates in the two-qubit space, including ${\sqrt{X}}_{Q1}$ (**f**), ${\sqrt{X}}_{Q2}$ (**g**), ${\sqrt{Y}}_{Q1}$ (**h**) and ${\sqrt{Y}}_{Q2}$ (**u**). **j**, SPAM error matrix of the measured two qubits estimated from GST, using the same method as used in^52^. **k**, The PTM of the standard $\sqrt{{\rm{SWAP}}}$ based on an isotropic Heisenberg exchange Hamiltonian. **l**, The experimentally measured PTM, ${M}_{\exp }$, obtained from GST. **m**, The theoretical PTM, *M*_the_, of the $\sqrt{{\rm{SWAP}}}$-style gate obtained by fitting the experimentally measured PTM, ${M}_{\exp }$, with a PTM generated by Eq. (28) in Supplementary Information VII (the fitted parameters are given there). The Hamiltonian Eq. (28) includes effects of spin–orbit coupling that are left out in the Hamiltonian of Eq. (2) of the main text. The fidelity of the $\sqrt{{\rm{SWAP}}}$-style gate is obtained from $F=\frac{1}{d+1}({\rm{Tr}}[{M}_{{\rm{the}}}^{-1}{M}_{\exp }]/d+1)$, where *d* = 2^*N*^ is the dimension of the Hilbert space, and *N* refers to the number of qubits.

With a combination of single-qubit gates and the SWAP-style gate, we prepare a Bell state and characterize it by quantum state tomography (QST). The pulse sequence shown in Fig. 3g is expected to produce a generalized Bell state $\left\vert \psi \right\rangle =\frac{1}{\sqrt{2}}(\left\vert {\mathrm{ST}}_{-}\right\rangle +{e}^{i\theta }\left\vert {\mathrm{T}}_{-}{\mathrm{S}}\right\rangle )$ between the two qubits, where *θ* is a single-qubit phase term, followed by single-qubit gates ($I,\,\sqrt{X},\,\sqrt{Y}$) applied to both qubits to achieve basis changes before measurement along the *z* axis. The density matrix of the Bell state formed by Q1–Q2 is shown in Fig. 3h, and the acquired fidelity and concurrence of all the neighbouring qubit pairs are shown in Fig. 3i (see Extended Data Fig. 8 for details). The fidelities are in the range of 73(1)–90(1)% and the concurrence ranges from 21(4)% to 64(4)%. Concurrence is a measure of the entanglement between two qubits, which ranges from 0 (no entanglement) to 1 (maximal entanglement). Therefore, the measured concurrence for all the qubit pairs demonstrates that the implemented $\sqrt{{\rm{SWAP}}}$ gate can generate entanglement between qubits. To evaluate the performance of the $\sqrt{{\rm{SWAP}}}$ gate, we perform two-qubit GST on Q1 and Q2. By fitting the result to a theoretical model, a gate fidelity of ~80% is obtained (see Extended Data Fig. 9 and Supplementary Note VIII for details).

## Quantum circuit implementation

**Fig. 4:**
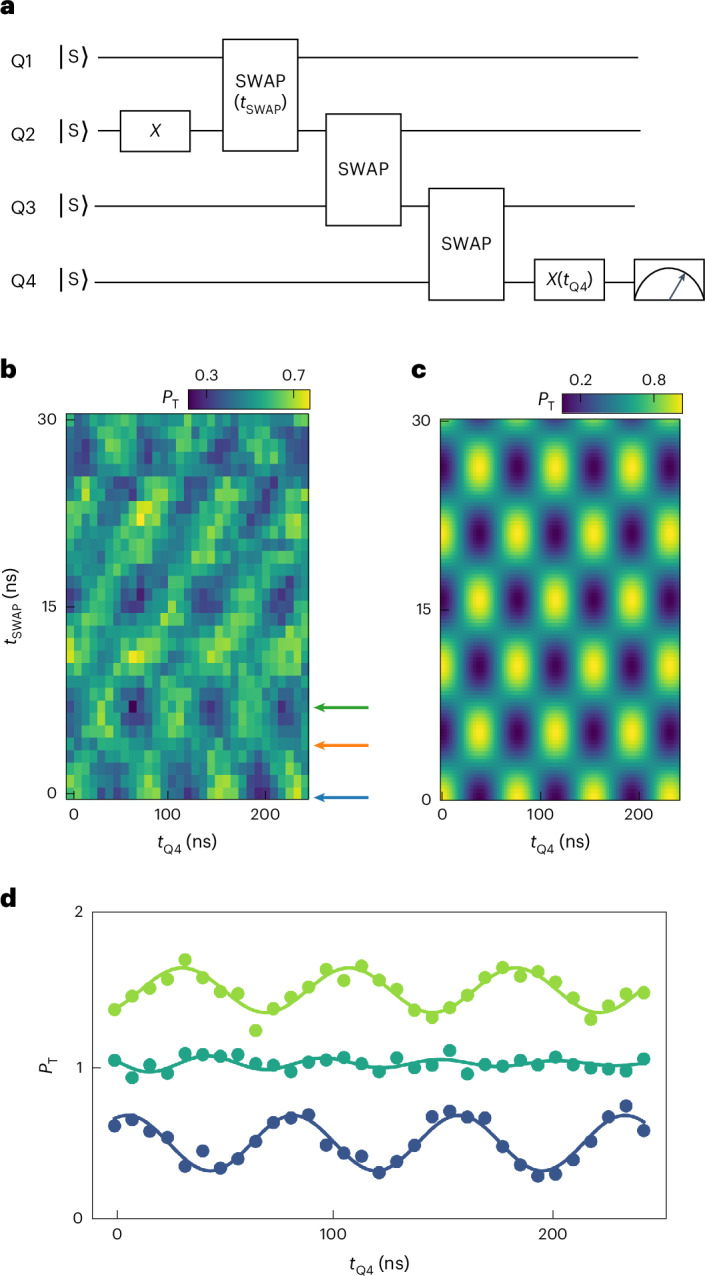
Implementation of a quantum circuit for entanglement generation and quantum state transfer. **a**, Quantum circuit with all the qubits initialized into the singlet. An *X* gate (32 ns) rotates Q2 into a triplet state, and a SWAP interaction for a variable time *t*_SWAP_ periodically produces entanglement between Q1 and Q2. Two subsequent SWAP gates (30 ns and 24 ns) transfer the state of Q2 to Q4 and a final single-qubit rotation of Q4 for a variable time *t*_Q4_ is followed by Q4 readout. The delay time between each quantum gate is set to zero. For more details, see Supplementary Note VI. **b**,**c**, Experimental (**b**) and numerical (**c**) results after running the quantum circuit shown in **a**, with triplet probabilities *P*_T_ of Q4 shown as a function of *t*_SWAP_ and *t*_Q4_. The coloured arrows in **b** show the positions of the linecuts in **d**. **d**, Linecuts from **a** showing triplet probabilities *P*_T_ of Q4 as a function of control time *t*_Q4_, with *t*_SWAP_ = 0, 4 and 7 ns (from bottom up). The data are vertically shifted by 0.5 for clarity. The 95% confidence intervals based on statistical fluctuations are smaller than the data points. All the data above are measured at *B* = 10 mT.

Finally, using a combination of the single- and two-qubit gates demonstrated above, we aim to implement a quantum circuit designed to create and distribute an entangled state across the array, as shown in Fig. 4a. The experimental results are shown in Fig. 4b, where the single-qubit oscillations of Q4 as a function of *t*_Q4_ are modulated in phase by *t*_SWAP_, resulting in a chequerboard pattern. The underlying mechanism is that the state of Q2 oscillates as a function of *t*_SWAP_, as quantum information is periodically exchanged between Q1 and Q2. Consequently, the state of Q4, following the quantum state transfer, also oscillates with *t*_SWAP_. Where the evolution of Q4 changes phase, *t*_SWAP_ corresponds to the duration of a $\sqrt{{\rm{SWAP}}}$ operation (modulo an integer number of SWAP operations), at which point maximal entanglement between Q1 and Q2 is expected. When two qubits are maximally entangled, the density matrix of each qubit by itself becomes fully mixed. At this point, the measured *P*_T_ of Q4 should remain constant and not oscillate as a function of *t*_Q4_. This is indeed what we observe in Fig. 4d, where we show the linecuts from Fig. 4b. A trace without apparent oscillations is observed between two sets of out-of-phase oscillations of Q4, as expected. The same features are seen in Fig. 4c, which shows the ideally expected chequerboard pattern obtained from numerical simulations of the protocol of Fig. 4a, assuming perfect initialization, operations and readout.

We note the chequerboard pattern is quite robust to errors in the SWAP gates. Small errors will merely change the contrast of the pattern; for large SWAP errors, the alternating rows are no longer equal in height. However, when the initialization of Q1 or Q2 leads to superposition states with a *y*-axis component (and assuming perfect SWAP gates), the pattern acquires a tilt. In this case, the rotation angle of the final *x*-axis rotation needed to maximize or minimize *P*_T_ is no longer exactly 0 or π but depends on the *y*-axis component of Q4 (and hence also on *t*_SWAP_) after the sequence of SWAP operations. Looking closely, the blue and green oscillations in Fig. 4d are not perfectly out of phase with each other, and the data in Fig. 4b show weak diagonal features not seen in the numerical simulations. These point at the imperfect initialization of Q1 or Q2.

We also characterize the remote Bell state of Q1 and Q4 by performing QST. The experiment was performed at *B* = 5 mT and the quantum circuit is similar except Q1 was initialized into a triplet instead of Q2. The resulting Bell state fidelity is 75(2)% and the concurrence is 22(4)%. Compared with the concurrence of the Bell state of Q1–Q2 before state transfer, which is 90(1)%, the remote entanglement is reduced by the transfer process of two consecutive SWAP gates.

## Conclusions

We have experimentally demonstrated initialization, readout and universal control of four singlet–triplet (S–T_−_) qubits in a 2 × 4 germanium quantum dot array. By using RB and QST, we obtain average single-qubit gate fidelities of 99.49(8)–99.84(1)% and Bell state fidelities of 73(1)–90(1)% for all the nearest qubit pairs. For the $\sqrt{{\rm{SWAP}}}$ gate, we estimate a gate fidelity of ~80% by fitting the GST result to a theoretical model. Furthermore, through independent control of the exchange interactions between any pair of neighbouring spins across the device, we are able to implement a quantum circuit that spans the entire array, yielding remote entanglement of two singlet–triplet qubits with a Bell state fidelity of 75(2)% and a concurrence of 22(4)%.

With four universally controlled qubits in a bilinear array, these results put baseband-controlled singlet–triplet spin qubits in germanium firmly on the map as a potential candidate for large-scale quantum computing. In future experiments, the two-qubit gate fidelity must be increased to allow fault-tolerant quantum computation. The gate fidelities can be potentially improved by suppressing low-frequency noise using feedback control or pulse optimization^29,54,55^ and by a more detailed modelling of the effects of spin–orbit interaction. Additionally, other types of two-qubit gates such as the CZ gate for S–T_−_ qubits can be investigated. Additional improvements can be reached if the tunnel barriers are more tunable, which can be achieved by depositing the barrier gates either before^13,15^ or together with^56^ the plunger gates. Moreover, with programmable control of exchange interactions in the array, this spin ladder can also be used for analogue simulation of a wealth of rich physical phenomena such as quantum magnetism^57^.

## Methods

### Device and set-up

The device was fabricated on a Ge/SiGe heterostructure with a strained germanium quantum well positioned 55 nm below the semiconductor–dielectric interface. The ohmic contacts were made by diffusing the aluminium into the heterostructure during annealing. The screening gates (3/17 nm, Ti/Pd), plunger gates (3/27 nm, Ti/Pd) and barrier gates (3/37 nm, Ti/Pd) were made in three metalization layers, which are all separated by 5-nm-thick layers of Al_2_O_3_ grown by atomic layer deposition.

The device is thermally anchored to the mixing chamber of a dilution refrigerator with a base temperature of around 10 mK. All the control electronics are at room temperature, which connect the device via 50 d.c. lines and 24 a.c. lines in total. The d.c. and a.c. signals are combined using bias tees on the printed circuit board with a resistor–capacitor time constant of 100 ms to apply both signals to the same gate of the device. For baseband pulses, a compensation pulse to the gate is applied to make the d.c. offset over the whole measurement cycle equal to zero, which mitigates the charging effects in the bias tees. Direct current signals are produced by custom-built battery-powered d.c. voltage sources and are fed through a matrix module—a breakout box with filters inside—to the Fisher cables of the fridge. Alternating signals are produced by six Keysight M3202A modules which are connected directly to the coaxial lines in the fridge. The output digital filter of the arbitrary waveform generator channels is set to the antiringing filter mode to suppress ringing effects in the baseband pulses. For the filters in the lines, we use common-mode ferrite chokes at room temperature to filter low-frequency noise (10 kHz–1 MHz) in the ground of a.c. lines and use resistor–capacitor filters (*R* = 100 kΩ, *C* = 47 nF for normal gates, *R* = 470 Ω, *C* = 270 pF for the ohmic contacts) and copper-powder filters that are mounted on the cold finger attached to the mixing chamber plate to filter high-frequency noise in d.c. lines. A detailed figure of the measurement set-up is shown in Supplementary Note I.

The sensing dots are measured using radiofrequency (RF) reflectometry with working frequencies of 179, 190, 124 and 158 MHz for sensors S_TL_, S_BL_, S_TR_ and S_BR_, respectively. Tank circuits are formed by NbTiN inductors mounted on the printed circuit board and the spurious capacitance of the bonding wires and metal lines on the board and chip. We apply RF signals using custom-built RF generators and combine them into a single coaxial line at room temperature using a power combiner (ZFSC-3-1W-S+). The signal is attenuated at each plate in the dilution refrigerator and passes through a directional coupler (ZEDC-15-2B) at the mixing chamber to reach the device. The signal reflected from the device goes through the same directional coupler and is then amplified with a CITLF3 cryogenic amplifier at the 4 K plate. At room temperature, the signal is amplified again and demodulated by custom-built in-phase and quadrature mixers. The demodulated signal is sent to a Keysight M3102A module to convert analogue readout signals to digital signals. We use d.c. blocks to reduce low-frequency noise (<10 MHz) in the RF lines. The d.c. block inside the refrigerator blocks the d.c. signal on the inner conductor (PE8210) while the ones at room temperature block that on both the inner and outer conductor (PE8212). To suppress high-frequency noise in the reflected signal, we use a low-pass filter (SBLP-300+) at room temperature.

### Initialization, control and readout

In the experiment, we repeatedly perform single-shot readout cycles to obtain singlet or triplet probabilities. The integration time for each single-shot readout is around 10–40 μs, depending on the signal-to-noise ratio and triplet relaxation time during measurements. To compensate for the drift of the sensor signal, we use a reference readout segment before each measurement sequence^12^. For some datasets, we adjust the single-shot readout threshold by postprocessing instead of through a reference segment. In postprocessing, we collect a histogram of 500–4,000 shots for each data point based on which we set the threshold to analyse those shots. In this way, the sensor drift between data points is mostly filtered out.

A typical pulse for single-qubit control can include initialization (20 μs), reference readout (20 μs), initialization (20–50 μs), control (30–3,000 ns) and readout (20 μs). A ramp-in time of 20 ns between initialization and control is used to avoid diabatic errors. The position of initialization is in the (0,2) or (2,0) regime but deeper than the PSB readout point to ensure fast relaxation to the ground state. In single-qubit GST measurements, the gate set includes a null operation. To avoid the readout immediately following the initialization, a waiting time of 10 ns at a point in the (1,1) regime is added to ensure the data acquisition is done correctly. This may decrease the readout fidelity when the waiting point causes unwanted rotations of the qubit. For this reason, the waiting point was moved to the readout position in the two-qubit GST experiment. For multi-qubit initialization and control, we initialize all the qubits into the singlet simultaneously by ramping from (2,0) or (0,2) to a high detuning point in (1,1), except for the qubit to be subject to single-qubit control, which is pulsed directly to the zero detuning point. We also found that adding a brief precontrol segment after initialization at high detuning in (1,1) for all qubits (wait about 2 ns) can give a better initialization to singlets. This variation is used in some of the experiments on QST and GST.

For the qubit operation times we used in the measurements of RB, QST and GST, the typical values are summarized as follows:

- $\sqrt{X}$: 43.5 ns (Q1), 27.5 ns (Q2), 35 ns (Q3) and 25 ns (Q4)
- *H*: 65 ns (Q1), 40 ns (Q2), 56 ns (Q3) and 40 ns (Q4)
- $\sqrt{{\rm{SWAP}}}$: 13 ns (Q1–Q2), 16.5 ns (Q2–Q3) and 11 ns (Q3–Q4)

### Randomized benchmarking

In single-qubit RB, we use the native gates *I*, $\sqrt{X}$ and $\sqrt{Y}$ to compose the sequences of Clifford gates. At the end of each sequence, a rotation is applied to (ideally) bring the qubit back to its initial state, and the final qubit state is measured using PSB. Experimentally, the single-qubit *I* gate is implemented as a pulse segment with zero waiting time. The Clifford gate sequence length varies from 2 to 232, and there are in total 30 random sequences for each sequence length. Single-shot measurement of the tested qubit is repeated 1,000 times for each random sequence to obtain the singlet or triplet probability. The measured data are fitted to a function ${P}_{\mathrm{S}}=A{p}_{\mathrm{c}}^{N}+B$, where *p*_*c*_ is the depolarizing parameter, *A* and *B* are the coefficients that absorb the state preparation and measurement errors, and *N* is the number of Clifford operations in the sequence. The average Clifford infidelity can then be described as *r*_c_ = (*d* − 1)(1 − *p*_c_)/*d*, where *d* = 2^*n*^ is the dimension of the system and *n* is the number of qubits. For the single-qubit operations used here, there are on average 3.625 generators per Clifford composition (Extended Data Table 1). Therefore, the average gate fidelity is given by *F*_g_ = 1 − *r*_c_/3.625. The uncertainties in the reported numbers represent 1 s.d. acquired from the curve fitting.

### Quantum state tomography

The density matrix of a two-qubit state can be expressed as $\rho =\mathop{\sum }\nolimits_{i = 1}^{16}{c}_{i}{M}_{i}$ where *M*_*i*_ are 16 linearly independent measurement operators, and the coefficients *c*_*i*_ are calculated from the expectation values *m*_*i*_ of the measurement operators using a maximum-likelihood estimate. In the experiment, we performed nine combinations of $\lbrace I,\,\sqrt{X},\,\sqrt{Y}\,\rbrace$ basis-change rotations on the two qubits and obtained the expectation values *m*_*i*_ by determining the joint two-qubit probabilities. To do so, we performed 500 single-shot measurements per sequence, and repeated the whole experiment 3–5 times. After that, the measured probabilities were converted to the estimated actual two-spin probabilities by removing the state preparation and measurement (SPAM) errors.

The SPAM matrix was measured by aiming to initialize two qubits into $\left\vert {\mathrm{SS}}\right\rangle ,\,\left\vert {\mathrm{ST}}_{-}\right\rangle ,\,\left\vert {\mathrm{T}}_{-}{\mathrm{S}}\right\rangle$ and $\left\vert {\mathrm{T}}_{-}{\mathrm{T}}_{-}\right\rangle$, and repeatedly measuring the two-qubit states in a single-shot manner. Then we use the relationship **P**_*M*_ = *M*_SPAM_**P**, where **P**_*M*_ are the measured two-qubit probabilities, *M*_SPAM_ is the SPAM matrix, and **P** are the actual two-qubit probabilities. We note this relationship works when the initialization error is negligible compared with the readout error, or it would cause miscorrections in the results.

Single-shot readout of two-qubit states was implemented differently for different qubit pairs. For Q1 and Q2, we first measure Q1 with an integration time of 20 μs while maintaining Q2 in the symmetry condition but with *δ*v*b*_26_ = − 60 mV to preserve its state. Next Q2 is measured. This method uses the same sensor for PSB readout of both Q1 and Q2, and therefore the two measurements have to be done sequentially. For Q2–Q3 and Q3–Q4, we performed SWAP gates to transfer the qubit information to Q1 and Q4, and the two qubits were measured simultaneously using two sensors on both sides. Also for the characterization of the remote Bell state Q1–Q4, the qubits Q1 and Q4 were measured simultaneously using the two sensors on both sides (after possible single-qubit rotations to change basis).

The single-qubit rotations before the final measurement were performed sequentially. Hence, the time between the $\sqrt{{\rm{SWAP}}}$ gate and the single-qubit gate of the second qubit can be dependent on any single-qubit operation being applied to the first qubit. These different times would cause different phase accumulations on the second qubit. To solve this problem, we use a waiting time as long as the longest qubit operation time of the first qubit before performing the basis-change rotation of the second qubit. This ensures the phase of the second qubit is consistent throughout the whole experiment (Extended Data Fig. 8a).

The Bell state fidelity is obtained from the experimentally obtained density matrix *ρ*_exp_ and the ideally expected density matrix, *ρ*_ideal_, and $F={\rm{Tr}}(\sqrt{\sqrt{{\rho }_{{\rm{ideal}}}}{\rho }_{\exp }\sqrt{{\rho }_{{\rm{ideal}}}}})$. The phase *θ* of the ideal Bell state $\left\vert \psi \right\rangle =\frac{1}{\sqrt{2}}(\left\vert {\mathrm{ST}}_{-}\right\rangle +{e}^{i\theta }\left\vert {\mathrm{T}}_{-}{\mathrm{S}}\right\rangle )$ is used as a fitting parameter to incorporate additional (fixed and predictable) single-qubit phase rotations before and after the $\sqrt{{\rm{SWAP}}}$ gate. The fitted *θ* for the Bell states Q1–Q2, Q2–Q3, Q3–Q4 and Q1–Q4 are 0.717, −0.614, −2.718 and 2.507, respectively. We note the non-ideal pulse effect between the concatenated single-qubit gate and the $\sqrt{{\rm{SWAP}}}$ gate may also result in other types of single-qubit rotations (Extended Data Fig. 9), which is not incorporated and can contribute to errors in the Bell state preparation. The uncertainties in the reported numbers are the standard deviations calculated from 2,000 bootstrap resampling iterations of the single-shot readout data for both the SPAM matrix and *P*_M_.

## Online content

Any methods, additional references, Nature Portfolio reporting summaries, source data, extended data, supplementary information, acknowledgements, peer review information; details of author contributions and competing interests; and statements of data and code availability are available at 10.1038/s41565-024-01817-9.

## Supplementary information

Supplementary Information Supplementary Notes I–VIII and Figs. 1–6.

## Figures and Tables

**Extended Data Table 1:**
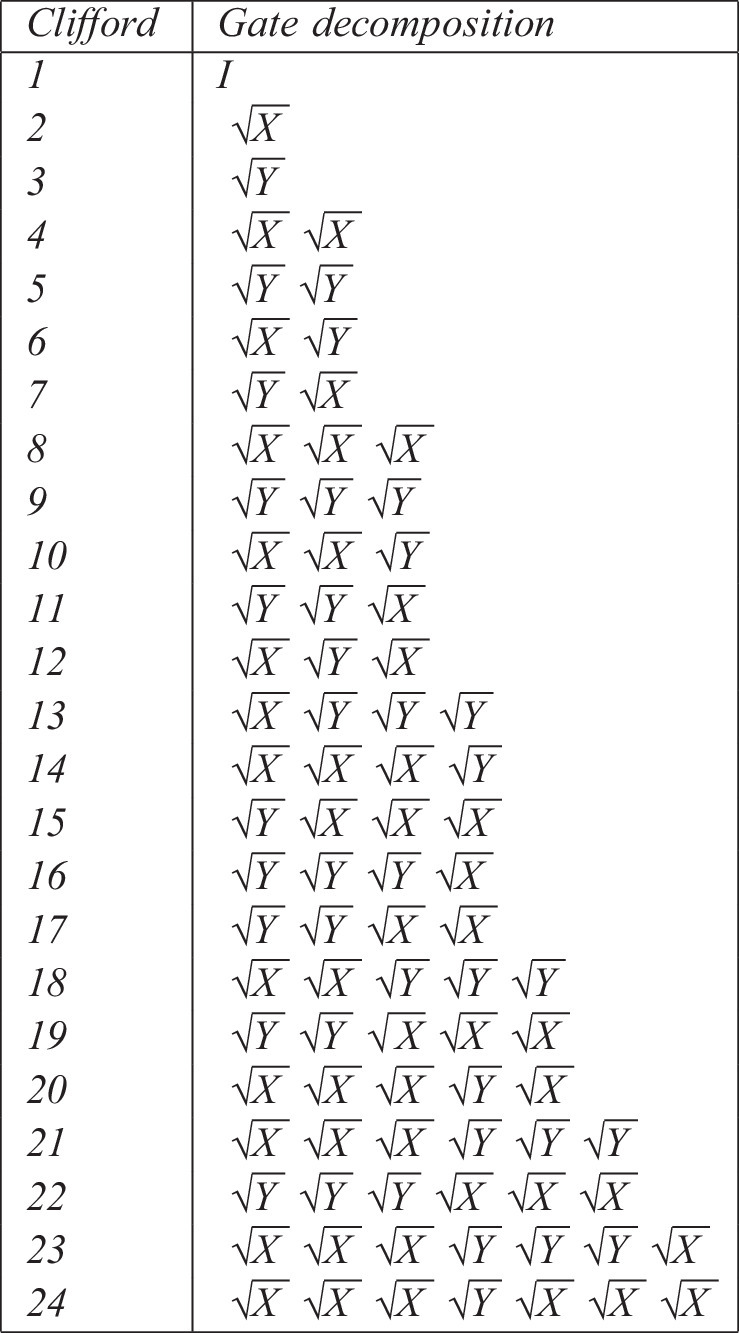
Single-qubit Clifford gate decomposition

.

## Data Availability

The data supporting this work are available on Zenodo via 10.5281/zenodo.12801188 (ref. ^58^).

## Code availability

The codes used for data processing and numerical simulation are available on Zenodo via 10.5281/zenodo.12801188 (ref. ^58^).
