# Supplementary material for: Universal control of four singlet–triplet qubits
Source: Nat Nanotechnol. 2024 Oct 31;20(2):209–15. doi: 10.1038/s41565-024-01817-9 (PMC11835736; doi:10.1038/s41565-024-01817-9)
Supplement: Supplementary file 1 — Supplementary Notes I–VIII and Figs. 1–6. [file 41565_2024_1817_MOESM1_ESM.pdf]

nature nanotechnology

Supplementary information https://doi.org/10.1038/s41565-024-01817-9

Universal control of four singlet–triplet qubits

In the format provided by the authors and unedited

This supplementary information includes:

- Supplementary Note I Experimental setup
- Supplementary Note II Virtual gate matrix
- Supplementary Note III Asymmetry in measured qubit energy spectrum
- Supplementary Note IV Additional data of $S - T_{-}$ oscillations
- Supplementary Note V Additional data of SWAP operations
- Supplementary Note VI Pulse scheme and calibration for quantum state transfer
- Supplementary Note VII A theoretical model for the SWAP operation
- Supplementary Note VIII Gate set tomography of the single- and two-qubit gate

I. EXPERIMENTAL SETUP

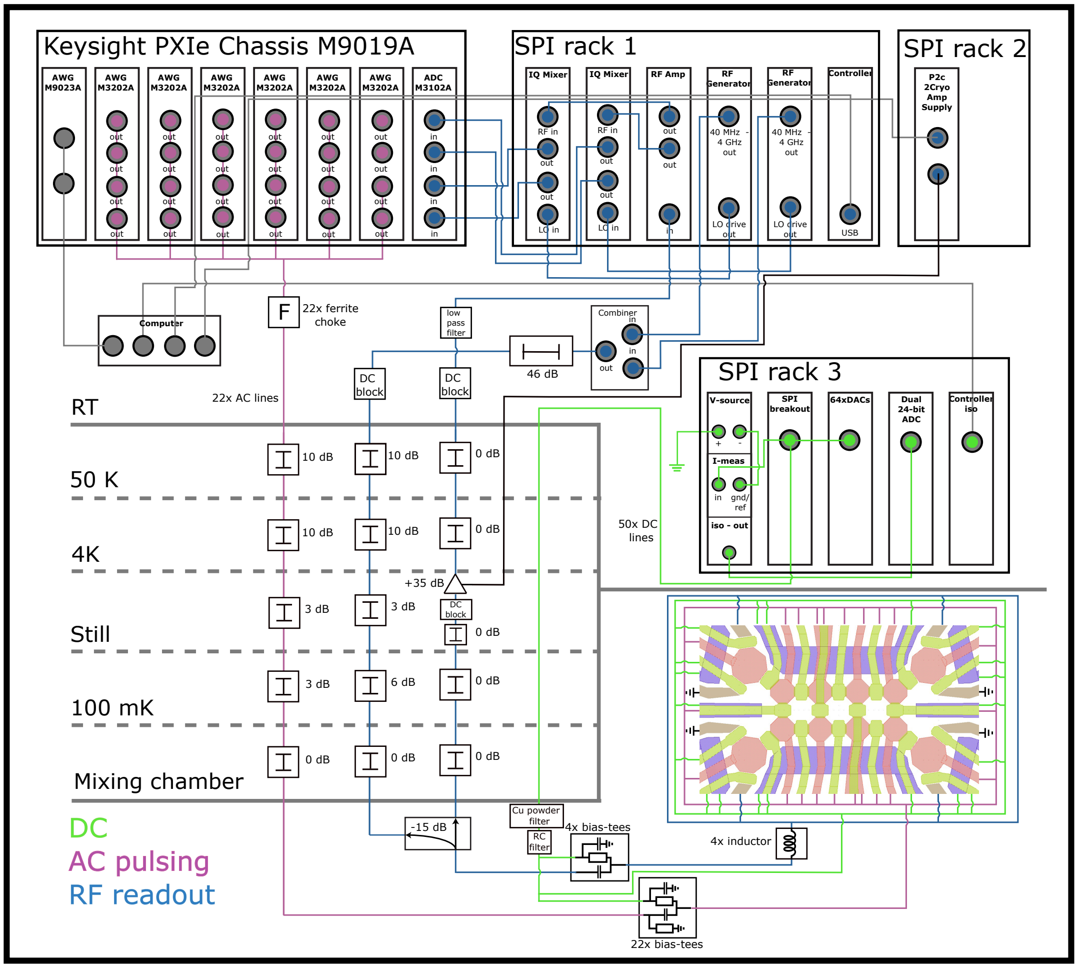

Supplementary Fig 1. Measurement circuit for the device. The DC and AC control lines as well as radio-frequency (RF) readout lines are fed from room-temperature instruments to the device at a base temperature of the dilution refrigerator through the cables and various electronic components shown in the figure. The room-temperature instruments include three custom-built SPI racks for supplying DC voltages and RF readout signals, and one Keysight PXIe Chassis for AC pulsing (arbitrary waveform generator, AWG) and data acquisition (analog-to-digital converter, ADC). The on-board bias-tees used for combining RF readout signals with DC voltages have an R=5 kΩ resistor, a C=100 pF capacitor to ground and a C=100 pF capacitor at the AC input; the one used for combining voltage pulses with DC signals has an R=1 MΩ resistor and a C=100 pF capacitor to connect the DC signal, and an R=100 kΩ resistor and a C=100 nF capacitor to connect the AC signal.

II. VIRTUAL GATE MATRIX

As mentioned in the main text, we use virtualized gates to independently control the chemical potential in each quantum dot. An example of the virtual gate matrix we used is as follows:

$$
\begin{pmatrix} \mathrm{vP}_1 \\ \mathrm{vP}_2 \\ \mathrm{vP}_3 \\ \mathrm{vP}_4 \\ \mathrm{vP}_5 \\ \mathrm{vP}_6 \\ \mathrm{vP}_7 \\ \mathrm{vP}_8 \\ \mathrm{vb}_{12} \\ \mathrm{vb}_{23} \\ \mathrm{vb}_{34} \\ \mathrm{vb}_{56} \\ \mathrm{vb}_{67} \\ \mathrm{vb}_{78} \\ \mathrm{vb}_{15} \\ \mathrm{vb}_{26} \\ \mathrm{vb}_{37} \\ \mathrm{vb}_{48} \end{pmatrix}
=
\begin{pmatrix}
1 & 0.3 & 0.1 & 0.05 & 0.35 & 0.2 & 0.05 & 0.02 & 0.4 & 0.08 & 0 & 0.05 & 0.05 & 0 & 0.4 & 0.15 & 0 & 0 \\
0.15 & 1 & 0.3 & 0.05 & 0.05 & 0.25 & 0.05 & 0 & 0.3 & 0.2 & 0 & 0.05 & 0.1 & 0 & 0.1 & 0.2 & 0 & 0 \\
0.05 & 0.25 & 1 & 0.25 & 0 & 0.1 & 0.35 & 0.05 & 0 & 0.15 & 0.06 & 0 & 0.15 & 0.12 & 0 & 0 & 0.08 & 0.07 \\
0.02 & 0.05 & 0.35 & 1 & 0.02 & 0 & 0.15 & 0.35 & 0 & 0.05 & 0.06 & 0 & 0 & 0.11 & 0 & 0 & 0.03 & 0.28 \\
0.15 & 0.15 & 0.03 & 0.02 & 1 & 0.25 & 0.05 & 0.03 & 0.1 & 0.05 & 0 & 0.2 & 0.05 & 0.03 & 0.3 & 0 & 0.02 & 0 \\
0.1 & 0.2 & 0.1 & 0.05 & 0.2 & 1 & 0.2 & 0.05 & 0.1 & 0.1 & 0.05 & 0.1 & 0.25 & 0 & 0.1 & 0.1 & 0 & 0 \\
0.05 & 0.15 & 0.25 & 0.1 & 0.08 & 0.22 & 1 & 0.15 & 0 & 0.1 & 0.02 & 0.05 & 0.25 & 0.28 & 0.05 & 0 & 0.05 & 0.05 \\
0 & 0.03 & 0.2 & 0.25 & 0.03 & 0.05 & 0.45 & 1 & 0 & 0.02 & 0.025 & 0.02 & 0.05 & 0.4 & 0 & 0 & 0.03 & 0.22 \\
0 & 0 & 0 & 0 & 0 & 0 & 0 & 0 & 1 & 0 & 0 & 0 & 0 & 0 & 0 & 0 & 0 & 0 \\
0 & 0 & 0 & 0 & 0 & 0 & 0 & 0 & 0 & 1 & 0 & 0 & 0 & 0 & 0 & 0 & 0 & 0 \\
0 & 0 & 0 & 0 & 0 & 0 & 0 & 0 & 0 & 0 & 1 & 0 & 0 & 0 & 0 & 0 & 0 & 0 \\
0 & 0 & 0 & 0 & 0 & 0 & 0 & 0 & 0 & 0 & 0 & 1 & 0 & 0 & 0 & 0 & 0 & 0 \\
0 & 0 & 0 & 0 & 0 & 0 & 0 & 0 & 0 & 0 & 0 & 0 & 1 & 0 & 0 & 0 & 0 & 0 \\
0 & 0 & 0 & 0 & 0 & 0 & 0 & 0 & 0 & 0 & 0 & 0 & 0 & 1 & 0 & 0 & 0 & 0 \\
0 & 0 & 0 & 0 & 0 & 0 & 0 & 0 & 0 & 0 & 0 & 0 & 0 & 0 & 1 & 0 & 0 & 0 \\
0 & 0 & 0 & 0 & 0 & 0 & 0 & 0 & 0 & 0 & 0 & 0 & 0 & 0 & 0 & 1 & 0 & 0 \\
0 & 0 & 0 & 0 & 0 & 0 & 0 & 0 & 0 & 0 & 0 & 0 & 0 & 0 & 0 & 0 & 1 & 0 \\
0 & 0 & 0 & 0 & 0 & 0 & 0 & 0 & 0 & 0 & 0 & 0 & 0 & 0 & 0 & 0 & 0 & 1
\end{pmatrix}
\begin{pmatrix} \mathrm{P}_1 \\ \mathrm{P}_2 \\ \mathrm{P}_3 \\ \mathrm{P}_4 \\ \mathrm{P}_5 \\ \mathrm{P}_6 \\ \mathrm{P}_7 \\ \mathrm{P}_8 \\ \mathrm{b}_{12} \\ \mathrm{b}_{23} \\ \mathrm{b}_{34} \\ \mathrm{b}_{56} \\ \mathrm{b}_{67} \\ \mathrm{b}_{78} \\ \mathrm{b}_{15} \\ \mathrm{b}_{26} \\ \mathrm{b}_{37} \\ \mathrm{b}_{48} \end{pmatrix}
$$

III. ASYMMETRY IN MEASURED QUBIT ENERGY SPECTRUM

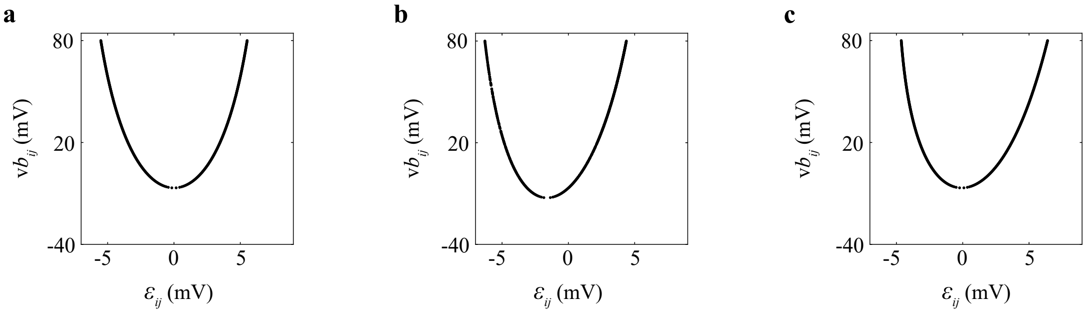

Supplementary Fig 2. **a**, Simulated parabola-like curve of the energy spectroscopy as a function of detuning ε_{ij} and barrier voltage vb_{ij} with a standard single-qubit Hamiltonian. **b**, Simulated asymmetrical curve by considering a detuning dependent g-factor. **c**, Simulated asymmetrical curve by considering a linear change of detuning offset as a function of vb_{ij}.

In Fig. 1 of the main text, we show energy spectrums of the $S-T_-$ qubits and mention that the asymmetry of the parabola-like curve may be caused by the detuning-dependent g-factor or an imperfect virtualization of the barrier gate. Here we do a numerical simulation to determine their effects. The results are shown in Supplementary Fig. 2a-c, which compares the standard energy spectrum (a) with the one with a detuning dependent g-factor (b) and that with imperfect virtualization (c). To numerically simulate these curves, we use the single-qubit Hamiltonian (13) and a relationship of $J = 2t^2U/(U^2 - \varepsilon^2)$. For the parameters, we use $U = 295$ GHz, $\overline{g} = 0.33$, $B = 10$ mT and $\Delta_{ST_-} = 16$ MHz. For the effect of g-factor change, we use a linear change of $\overline{g} = 0.19$-0.47 as a function of detuning, while for the effect of imperfect virtualization, we use a linear variation of detuning offset ε_0 as a function of the barrier gate voltage vb_{ij}, which changes in the range of -0.88 - 0.44 mV when vb_{ij} is changed from 80 to -40 mV. The variation of g-factor is a bit far away from the values reported in a similar device, where they report a change of 0.21-0.29 as a function of detuning [1]. But the 1% variation of ε_0 is plausible considering the imperfect virtualization. Still, the observed asymmetrical curve in the main text may result from their combined effects.

IV. ADDITIONAL DATA OF $S - T_-$ OSCILLATIONS

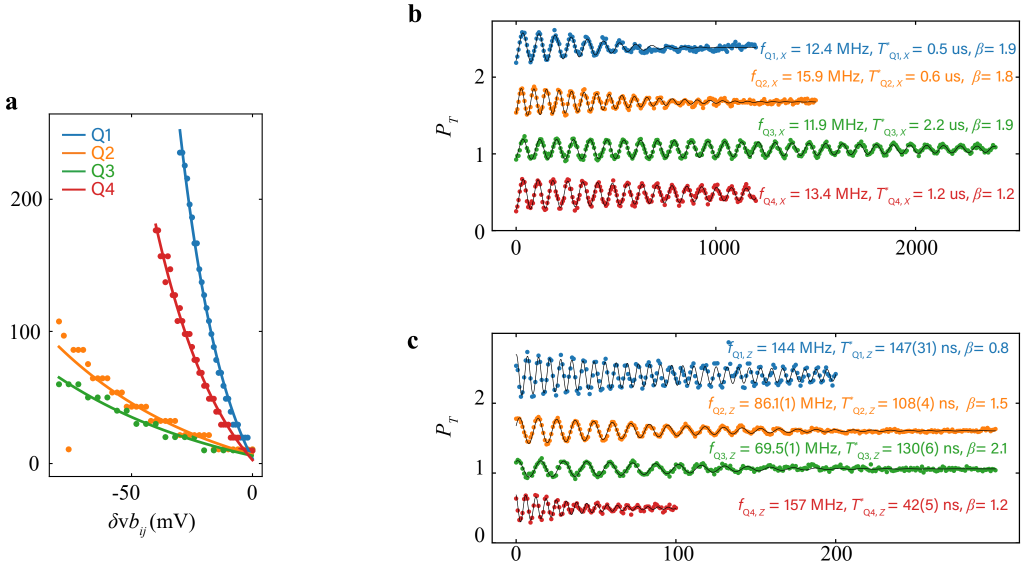

Supplementary Fig 3. **a**, Rotation frequency f_{ST_-} of each qubit as a function of δvb_{ij}, extracted from Extended Data Fig.2**g-j** using a FFT. The data points for Q2 and Q3 show discrete steps as the barrier gate voltage changes, which are caused by the FFT precision. The solid lines are fits of the data with an exponential function. **b,c**, Measured triplet probabilities P_T of long-time evolutions around the x- (**b**) and z-axis (**c**) for Q1-Q4 at B=10 mT. The data is vertically shifted for clarity. In **b**, the detuning is around 0 mV. In **c**, we take the data with the same detuning but with different barrier voltage pulses: $\delta vb_{15} = -20$ mV, $\delta vb_{26} = -80$ mV, $\delta vb_{37} = -80$ mV, and $\delta vb_{48} = -30$ mV. The barrier voltages are chosen to achieve the condition $J - \overline{E}_z \gg \Delta_{ST_-}$ depending on the gate tunability. Extracted parameters from the data fitting are in the inset, with the errors representing the 68% confidence intervals obtained from fitting.

The additional data here is collected at B= 10 mT, under similar conditions as the data shown in Extended Data Fig.2.

Supplementary Fig. 3a summarizes how f_{ST_-} can be tuned via δvb_{ij}. As discussed in the main text, the outer two barrier gates vb_{15} and vb_{48} have a stronger effect on the corresponding J_{ij} than the inner barrier gates vb_{26} and vb_{37}. This may possibly be explained by resist residues below the inner barrier gates, which are fabricated in the last step [2], and by the different fan-out routing for the outer barrier gates (see Fig.1a,b in the main text).

The dephasing time under free evolution, which is traditionally termed T_2^*, is an important metric for assessing the qubit quality. Since the qubit rotations around both the x-axis and the z-axis are the result of free evolution, we introduce T_x^* and T_z^* to describe the corresponding dephasing times. Supplementary Fig. 3b,c show the measured damped oscillations of the qubits under x-axis and z-axis control. From the fits, we obtain a T_x^* of 0.5 - 2.1 μs and a T_z^* of 42(5) - 147(31) ns.

The measured values of T_x^* are slightly lower than previously reported values measured at $B = 1$ mT [3] under the same condition that B is parallel to the hole movement direction. This can be partly attributed to the larger magnetic field in panel b: Δ_{ST_-} scales with B [4, 5] and not only sets f_{ST_-} but also constitutes a proportionality factor between noise and f_{ST_-} fluctuations (see also Supplementary Information section VIII). The extracted x-axis rotation frequencies in Supplementary Fig. 3b reflect the values of Δ_{ST_-} for each qubit, which are around 11.9-15.9 MHz, much larger than the results reported at $B = 1$ mT [3]. This confirms that Δ_{ST_-} is stronger in the present experiment than in the previous work at 1 mT. The small variation in Δ_{ST_-} and in the measured average g-factors suggests a fairly homogeneous spin-orbit coupling in this device. The extracted parameter β also has a big variation among qubits, and considering the variations in T_x^* are also large, especially for the data at $B = 10$ mT, these qubits may suffer from spatially dependent charge noise or inhomogeneous hyperfine-induced dephasing due to dot size differences [6].

We also observe that T_z^* is roughly an order of magnitude smaller than T_x^* in Supplementary Fig. 3b. Possibly this is due to the fact that fluctuations in the tunnel barrier translate to fluctuations in J, which couple in directly during z-axis evolution but only to second order for x-axis evolution [7, 8]. Additionally, the increased curvature of

the singlet branch for larger J implies a higher sensitivity to detuning noise. The variations in J thus may contribute to the spread of the T_z^* values we obtained in Supplementary Fig. 3c.

V. ADDITIONAL DATA OF SWAP OPERATIONS

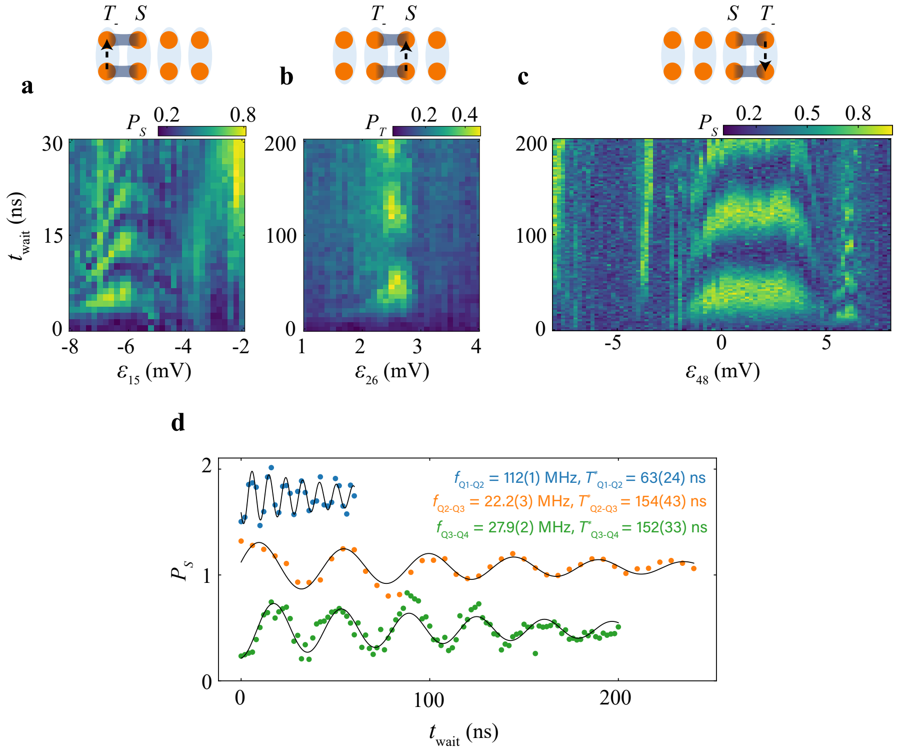

Supplementary Fig 4. **a-c**, The experimental results of SWAP oscillations measured at $B = 10$ mT, showing triplet probabilities P_T or singlet probabilities P_S as a function of operation time $t_{\rm wait}$ and the detuning voltage ε_{ij} for Q1-Q2 (**a**), Q2-Q3 (**b**) and Q3-Q4 (**c**). The initial states of two qubits (before the SWAP oscillations) are denoted on the top, and the qubit pair that is read out is indicated by the dashed arrow showing the readout pulse direction. **d**, Measured singlet probabilities P_S at $B = 10$ mT as a function of the evolution time $t_{\rm wait}$ at the center of the chevron patterns of the SWAP oscillations for each pair of qubits. Extracted parameters from the data fitting are in the inset, with errors representing the 68% confidence intervals. Notice the conditions are slightly different from the two-dimensional scanned data due to device tuning.

The additional data here is collected at B= 10 mT, under similar conditions as the data shown in Fig.4b of the main text.

Supplementary Fig. 4a-c shows SWAP oscillations of Q1-Q2, Q2-Q3, and Q3-Q4. For Q3-Q4, we scanned over a wide range of detuning and there are three sets of oscillations visible from left to right, where the rightmost corresponds to SWAP oscillations of Q3-Q4, the middle oscillations correspond to a single-qubit operation of Q4, and the leftmost is also a set of SWAP oscillations but with a very slow oscillation speed, which corresponds to a smaller interqubit coupling than the rightmost one (see the leftmost anticrossing in Fig. 3b of the main text). These features can all be qualitatively understood using Fig. 3b in the main text. However, to quantitatively model the oscillations, the contribution of anisotropic exchange couplings may be needed.

The oscillation frequencies in the middle of the chevron patterns are in the range of 22.2(3) - 112(1) MHz, corresponding to a $\sqrt{\rm SWAP}$ (entangling) gate with durations of just 2.2 ns to 11.3 ns, more than an order of magnitude faster than the entangling gate based on capacitive coupling [9, 10]. To determine the dephasing times of the SWAP oscillations, we collect data in the middle of the chevron patterns, as shown in Supplementary Fig. 4d, and fit them with the same function as used for single-qubit oscillations. The extracted dephasing times are between 63(24) and 154(43) ns. An increased quality of SWAP oscillations can be observed in the Extended Fig. 3b, of which the data was collected at B = 5 mT in a separate cool-down of the device. The dephasing times may be further increased by executing the SWAP oscillations at the symmetry points of the detuning of each qubit, which requires a stronger tunability of the exchange interactions using the barrier gates.

VI. PULSE SCHEME AND CALIBRATION FOR QUANTUM STATE TRANSFER

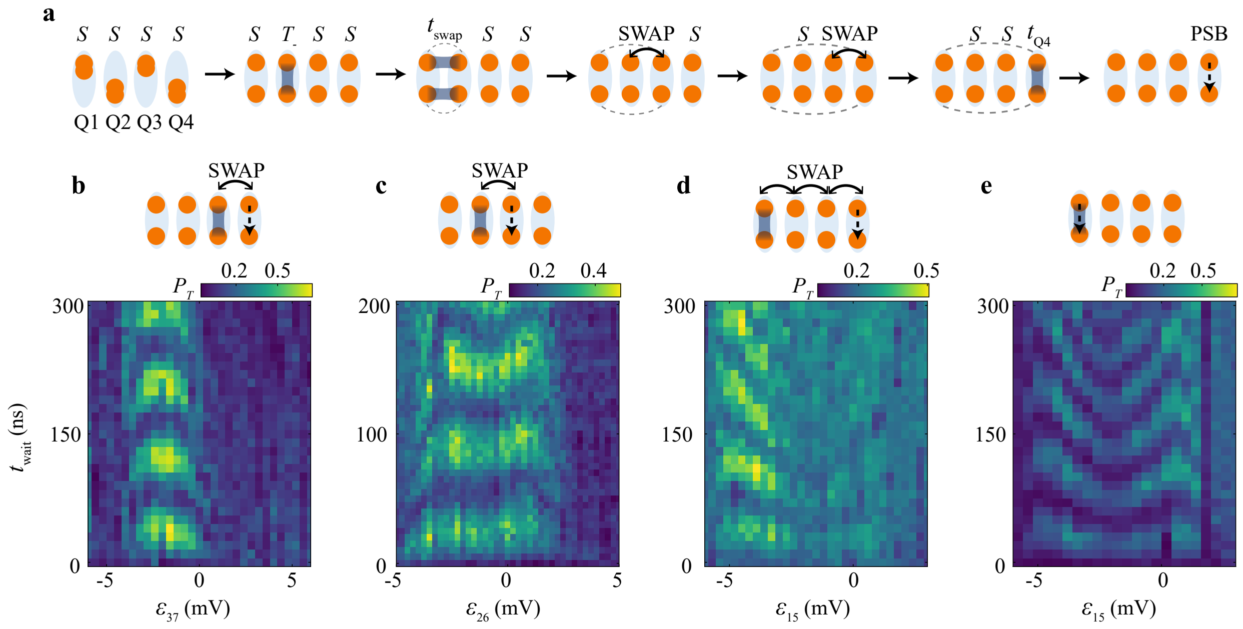

Supplementary Fig 5. **a**, Schematic representation of the different steps in the quantum-state-transfer experiment of Fig. 4 of the main text. The gray dashed curves indicate potential entanglement between two qubits. The black curved double-arrow refers to a SWAP gate that is intended to transfer information from one qubit to the next. **b**, Measured triplet probabilities of Q4 as a function of waiting time t_{wait} and the detuning of Q3, ε_{37}. The inset illustrates that we control Q3 to perform an x-rotation with a time t_{wait} and subsequently perform a SWAP gate of Q3-Q4 and measure Q4 using PSB. **c**, Measured triplet probabilities of Q3 as a function of t_{wait} and detuning of Q2, ε_{26}. The inset illustration is similar to that in **b** but with single-qubit control of Q2, a subsequent SWAP gate of Q2-Q3 and PSB measurement of Q3. **d**, Measured triplet probabilities of Q4 as a function of t_{wait} and the detuning of Q1, ε_{15}. The inset shows that we perform an x-rotation of Q1 with a time t_{wait} followed by three consecutive SWAP gates to transfer the state of Q1 to Q2, Q3 and Q4, after which we measure Q4 using PSB. **d** has lower visibility on the right-hand side of the figure compared to its left-hand side, which can be attributed to a possible parameter shift of the consecutive SWAP gates by low-frequency charge noise after the left part was scanned. **e**, Direct readout of Q1 with the same single-qubit control as in **d**.

The detailed pulse scheme for collecting the data of quantum state transfer in Fig.4b of the main text is shown in Supplementary Fig. 5a. We initialize Q1-Q4 in the $\left(\begin{smallmatrix}2&0&2&0\\0&2&0&2\end{smallmatrix}\right)$ regime and then diabatically pulse to the $\left(\begin{smallmatrix}1&1&1&1\\1&1&1&1\end{smallmatrix}\right)$ regime, preserving four singlets. At this point, the detuning of Q1 and Q3 is set close to the (1,1)-(2,0) transition and that of Q4 close to the (1,1)-(0,2) transition in order to stay away from the $S-T_-$ anticrossing. This ensures that these three qubits remain in the singlet state. The detuning of Q2 is set to zero such that Q2 rotates around its x axis to $|T_-\rangle$. Then we pulse the detunings and barrier gates of Q1-Q2 to initiate a SWAP interaction for a duration t_{wait}, after which we transfer the state of Q2 to Q4 by performing sequential SWAP operations of Q2-Q3 and Q3-Q4. Finally, we pulse Q4 to zero detuning to kickstart single-qubit evolution around the x-axis for a duration t_{Q4}. For readout, we pulse Q1-Q4 into $\left(\begin{smallmatrix}1&1&1&0\\1&1&1&2\end{smallmatrix}\right)$ where we perform PSB readout of Q4. The detunings of Q1-Q3 are set to zero while Q4 is read out, but this will not affect the readout since Q4 is protected by a very large J in the readout window.

Before the quantum state transfer experiment, we confirm the operation of the individual SWAP gates by performing a single-qubit rotation of one qubit and measuring its state using the other after a SWAP gate. Some results are shown in Supplementary Fig. 5b and c, where we perform this test for Q3-Q4 and Q2-Q3, respectively. Supplementary Fig. 5d shows a result where Q1 is rotated and the measured qubit is Q4 following three consecutive SWAP gates from Q1 to Q4. A comparison of the direct readout of Q1 is shown in Supplementary Fig. 5e, with similar oscillations to that of Supplementary Fig. 5d at similar detuning voltages. These results demonstrate the SWAP gates are of sufficient quality to allow quantum state transfer across the entire array.

VII. A THEORETICAL MODEL FOR THE SWAP OPERATION

Holes confined in quantum dots are well described by the conventional Fermi-Hubbard model [11]. In the presence of a finite magnetic field and spin-orbit interaction, the Fermi-Hubbard model can be written as:

$$H_{\rm FH} = \sum_{i=1} \left[\mu_i n_i + \mu_B \boldsymbol{B} \cdot \tilde{\mathcal{G}}_i \boldsymbol{S}_i\right] + \sum_{i=1} \frac{\tilde{U}_i}{2} n_i(n_i - 1) + \sum_{\langle i,j \rangle} V_{ij} n_i n_j$$
$$+ \sum_{\sigma=\uparrow,\downarrow} \left(\tilde{t}_{ij} c^\dagger_{i,\sigma} c_{j,\sigma} + \text{h.c.}\right) + \sum_{\sigma=\uparrow,\downarrow} \left(\tilde{t}_{\text{SO},ij} c^\dagger_{i,\sigma} c_{j,\bar{\sigma}} + \text{h.c.}\right). \quad (1)$$

Here, h.c. denotes hermitian conjugate, $\bar{\sigma}$ the opposite spin state of σ, the operator $c^\dagger_{i,\sigma}$ ($c_{i,\sigma}$) creates (annihilates) a hole in QD i with spin $\sigma =\uparrow, \downarrow$, $n_i \equiv \sum_\sigma c^\dagger_{i,\sigma} c_{i,\sigma}$ is the charge number operator, and $\boldsymbol{S} = (S_{x,i}, S_{y,i}, S_{z,i})^T$ is the vector consisting of the spin matrices

$$S_{x,i} = \frac{\hbar}{2}(c^\dagger_{i,\uparrow} c_{i,\downarrow} + c^\dagger_{i,\downarrow} c_{i,\uparrow}) \quad (2)$$
$$S_{y,i} = -i\frac{\hbar}{2}(c^\dagger_{i,\uparrow} c_{i,\downarrow} - c^\dagger_{i,\downarrow} c_{i,\uparrow}) \quad (3)$$
$$S_{z,i} = \frac{\hbar}{2}(c^\dagger_{i,\uparrow} c_{i,\uparrow} - c^\dagger_{i,\downarrow} c_{i,\downarrow}). \quad (4)$$

The first line in Hamiltonian (1) describes the hole energies, i.e., μ_i denotes the chemical potential in QD i, $\tilde{U}_i$ is the Coulomb repulsion for doubly occupying the i-th QD, V_{ij} is the Coulomb repulsion of two holes occupying neighboring QDs i and j, μ_B is Bohr's magneton, and $\tilde{\mathcal{G}}_i$ is the quantum dot g-tensor. The second line describes the hopping terms between neighboring sites denoted by the spin-conserving tunnel matrix elements $\tilde{t}_{ij}$ and spin-non-conserving tunnel matrix elements $\tilde{t}_{\text{SO},ij}$. Note, that this notation is consistent with Refs. [12, 13] as $\tilde{t}_{ij}$ and $\tilde{t}_{\text{SO},ij}$ can be complex in general.

Single-qubit Hamiltonian

Considering only two neighboring quantum dots, the Hamiltonian can be simplified by transforming into the spin-orbit frame [13]. Intuitively, since the spin-orbit interaction only rotates the spin during tunneling, local spin rotations can always "unwind" the rotation. As a consequence of the spin-orbit interaction, the g-tensor of quantum dot i is rotated as $\tilde{\mathcal{G}}_i \to R_i \tilde{\mathcal{G}}_i \equiv \mathcal{G}_i$, and the tunnel coupling is renormalized as $t = \sqrt{|\tilde{t}|^2 + |\tilde{t}_{\text{SO}}|^2}$ [13].

Hamiltonian The respective Hamiltonian in the basis $\{S(2,0),\ S(0,2),\ S(1,1),\ T_-(1,1),\ T_0(1,1),\ T_+(1,1)\}$ of the spin-orbit frame reads

$$H_{\rm DQD} = \begin{bmatrix} U+\varepsilon & 0 & \sqrt{2}t & 0 & 0 & 0 \\ 0 & U-\varepsilon & \sqrt{2}t & 0 & 0 & 0 \\ \sqrt{2}t & \sqrt{2}t & 0 & \frac{\Delta E_x - i\Delta E_y}{\sqrt{2}} & \Delta E_z & -\frac{\Delta E_x + i\Delta E_y}{\sqrt{2}} \\ 0 & 0 & \frac{\Delta E_x + i\Delta E_y}{\sqrt{2}} & -\bar{E}_z & \frac{\bar{E}_x + i\bar{E}_y}{\sqrt{2}} & 0 \\ 0 & 0 & \Delta E_z & \frac{\bar{E}_x - i\bar{E}_y}{\sqrt{2}} & 0 & \frac{\bar{E}_x + i\bar{E}_y}{\sqrt{2}} \\ 0 & 0 & \frac{-\Delta E_x + i\Delta E_y}{\sqrt{2}} & 0 & \frac{\bar{E}_x - i\bar{E}_y}{\sqrt{2}} & \bar{E}_z \end{bmatrix}. \quad (5)$$

Here, we introduce the energy detuning $\varepsilon = \mu_1 - \mu_2$, and the average and difference in Zeeman energy for a fixed magnetic field direction, $2\hat{E}_\xi = \mu_B \boldsymbol{B}(\mathcal{G}_1 + \mathcal{G}_2)\hat{\xi}$ and $2\Delta E_\xi = \mu_B \boldsymbol{B}(\mathcal{G}_1 - \mathcal{G}_2)\hat{\xi}$, where $\hat{\xi}$ is the unit vector along axis $\xi = x, y, z$. We note that in our experiment we only have access to the energies and not the individual g-tensors due to the lack of access to a vector magnet.

Simulations To compute the single-qubit energy spectra in the main text (Fig. 1c-e), we use Hamiltonian (1) assuming only real tunnel matrix elements. Explicitly, we set the spin-conserving tunneling as $\tilde{t} = 1.2, 1.86, 2.4$ GHz, the spin-non-conserving tunneling as $\tilde{t}_{\text{SO}} = 10$ MHz, and $U = 300$ GHz, $B = |\boldsymbol{B}| = 10$ mT, $\bar{g} = |\mathcal{G}_1 + \mathcal{G}_2|/2 = 0.33$, $\Delta g = |\mathcal{G}_1 - \mathcal{G}_2|/2 = 0.008$, and with all other components of the g-tensors set to zero.

Effective qubit Hamiltonian To separate the spin dynamics from the charge dynamics, we now restrict ourselves to the subspace spanned by the spin states $\{S(1,1),\, T_-(1,1),\, T_0(1,1),\, T_+(1,1)\}$. This can be done either by block-diagonalizing via Schrieffer-Wolff perturbation theory [14] or by diagonalizing the singlet sector spanned by $\{S(2,0),$ $S(0,2),\, S(1,1)\}$ with a subsequent projection or block-diagonalization [13]. Regardless of the chosen method, the resulting Hamiltonian has the form of a generalized Heisenberg Hamiltonian

$$H_{\mathrm{DQD}} = \begin{bmatrix} J_0 & \frac{\Delta E_x - i\Delta E_y}{\sqrt{2}} & \Delta E_z & -\frac{\Delta E_x + i\Delta E_y}{\sqrt{2}} \\ \frac{\Delta E_x + i\Delta E_y}{\sqrt{2}} & -\bar{E}_z & \frac{\bar{E}_x + i\bar{E}_y}{\sqrt{2}} & 0 \\ \Delta E_z^* & \frac{\bar{E}_x - i\bar{E}_y}{\sqrt{2}} & 0 & \frac{\bar{E}_x + i\bar{E}_y}{\sqrt{2}} \\ \frac{-\Delta E_x + i\Delta E_y}{\sqrt{2}} & 0 & \frac{\bar{E}_x - i\bar{E}_y}{\sqrt{2}} & \bar{E}_z \end{bmatrix}, \tag{6}$$

where J_0 is the exchange interaction. In the regime of single dot occupation, $|t| \ll |U \pm \epsilon|$, the exchange interaction can be approximated by [14]

$$J = \frac{2t^2 U}{U^2 - \varepsilon^2}. \tag{7}$$

We observe that during the transformation $\Delta E_{x,y,z}$ is renormalized by the charge-state admixture as $\Delta E'_{x,y,z}$ [13, 15]. For simplicity and without impacting practical outcomes, we henceforth omit the primed notation.

Starting from Hamiltonian (6) we can now construct the qubit Hamiltonian. The $|0\rangle \equiv |S\rangle = S(1,1)$ qubit state is defined via the PSB readout mechanism. We find the second qubit state by diagonalizing the triplet sector spanned by $\{T_-(1,1),\, T_0(1,1),\, T_+(1,1)\}$. Fortunately, the transformation can be parameterized as $e^{-i\boldsymbol{v}\cdot\boldsymbol{S}^0/\hbar}$, where $\boldsymbol{v}$ is a real vector and $\boldsymbol{S}^0$ is a vector containing the spin-0 matrices

$$S_x^0 = \frac{1}{2}(S_{x,1} + S_{x,2}) \tag{8}$$

$$S_y^0 = \frac{1}{2}(S_{y,1} + S_{y,2}) \tag{9}$$

$$S_z^0 = \frac{1}{2}(S_{z,1} + S_{z,2}). \tag{10}$$

The $|1\rangle \equiv |T_-\rangle$ qubit state is then consequently given by the lowest-energy state. The final qubit Hamiltonian reads

$$\tilde{H}_{ST_-} = \begin{bmatrix} -J & \frac{\Delta_{ST_-} e^{i\phi_{ST_-}}}{2} \\ \frac{\Delta_{ST_-} e^{-i\phi_{ST_-}}}{2} & -\overline{E}_z \end{bmatrix}. \tag{11}$$

In lowest-order perturbation theory the exchange energy $J = J_0$, the average Zeeman splitting $\overline{E}_z = \sqrt{\bar{E}_x^2 + \bar{E}_y^2 + \bar{E}_z^2}$, and the spin-orbit coupling reads

$$\Delta_{\mathrm{SO}} e^{i\phi_{ST_-}} = \sqrt{2}\frac{\Delta E_z\left(\bar{E}_x^2 + \bar{E}_y^2\right) - \bar{E}_z(\Delta E_x \bar{E}_x + \Delta E_y \bar{E}_y)}{\overline{E}_z(\hat{E}_x - i\bar{E}_y)} + \sqrt{2}i\frac{\Delta E_x \bar{E}_y - \Delta E_y \bar{E}_x}{(\hat{E}_x - i\bar{E}_y)}. \tag{12}$$

To arrive at the single-qubit Hamiltonian in the main text, we apply the local phase transformation $U_\phi =$ $\exp(-i\phi_{ST_-}(|0\rangle\langle 0| - |1\rangle\langle 1|)/2)$

$$H_{ST_-} = U_\phi \tilde{H}_{ST_-} U_\phi^\dagger = \begin{bmatrix} -J & \frac{\Delta_{ST_-}}{2} \\ \frac{\Delta_{ST_-}}{2} & -\overline{E}_z \end{bmatrix}, \tag{13}$$

We remark that a more accurate Hamiltonian can be computed recursively using perturbation theory for a sufficiently large spectral gap [16]. We also remark that leakage outside the qubit subspace can be strongly suppressed using optimal control theory and Hamiltonian engineering.

Decoherence times

Charge noise and nuclear spin noise are ubiquitous in germanium semiconductor devices. Due to the low-frequency nature of both types of noise, they can be approximately modeled as quasistatic fluctuations of input parameters, giving rise to pure dephasing. Furthermore, we ignore in our analysis any energy relaxation mechanism, which is a good approximation for spin qubits [11]. Hamiltonian (13) under weak low-frequency noise thus becomes [17]

$$H_{ST_-} = \begin{bmatrix} -J - \delta J & \frac{\Delta_{ST_-}+\delta\Delta_x - i\delta\Delta_y}{2} \\ \frac{\Delta_{ST_-}+\delta\Delta_x + i\delta\Delta_y}{2} & -\overline{E}_z - \delta\overline{E}_z \end{bmatrix}, \tag{14}$$

where δ denotes fluctuations. Note that there are two off-diagonal contributions $\delta\Delta_x$ and $\delta\Delta_y$ that arise from the complex quantity $\Delta_{ST_-} e^{i\phi_{ST_-}}$. Assuming quasi-static noise (or low-frequency noise within the adiabatic approximation) the qubit resonance frequency is modulated by noise as follows

$$\hbar\omega_q = \sqrt{4(J-\overline{E}_z)^2 + \Delta_{ST_-}^2} \to \sqrt{4(J-\overline{E}_z+\delta J-\delta\overline{E}_z)^2 + (\Delta_{ST_-}+\delta\Delta_x)^2 + \delta\Delta_y^2}. \tag{15}$$

The $S-T_-$ qubit is operated in two regimes which we separately discuss.

Dephasing during rotations around the x-axis For single-qubit x-rotations, we operate at $J = \overline{E}_z$ and we can expand Eq. (15) up to first order

$$\hbar\omega_q^x = \Delta_{ST_-} + \delta\Delta_x + \mathcal{O}(\delta^2) \tag{16}$$

to find the dominating contributions. Assuming quasi-static Gaussian distributed noise, the decoherence time in the x-axis rotation regime is then given by

$$T_x^* = \frac{1}{\sqrt{2}\sigma_{\Delta_x}}, \tag{17}$$

where $\sigma_{\Delta_x}^2 = \langle\delta\Delta_x^2\rangle - \langle\delta\Delta_x\rangle^2$ is the standard deviation of the $\delta\Delta_x$ noise. Since $\Delta E_\xi \propto B$ and $\overline{E}_\xi \propto B$ for $\xi = x, y, z$, we expect $T_x^* \propto B^{-1}$ for an in-plane magnetic field [6, 18]. Therefore, operating at small magnetic fields is beneficial for x-gates.

Dephasing during rotations around the z-axis For single-qubit z-rotations, we operate at $|J-\overline{E}_z| \gg \Delta_{ST_-}$ and we can expand Eq. (15) up to first order

$$\hbar\omega_q^z = |J-\overline{E}_z+\delta J-\delta\overline{E}_z| + \mathcal{O}(\delta^2) \tag{18}$$

to find the dominating contributions. Assuming Gaussian distributed noise the decoherence time in the z-axis rotation regime is then given by

$$T_z^* = \frac{1}{\sqrt{2}\sigma_{J,\overline{E}_z}(1+c)}, \tag{19}$$

where $\sigma_{\sigma_{J,\overline{E}_z}}^2 = \langle(\delta J-\delta\overline{E}_z)^2\rangle - \langle(\delta J-\delta\overline{E}_z)\rangle^2$ is the standard deviation of the noise difference, $c = \mathrm{cov}(J,\overline{E}_z)/(\sigma_J\sigma_{\overline{E}_z})$ the noise correlation factor, $\mathrm{cov}(J,\overline{E}_z)$ the covariance, and σ_J ($\sigma_{\overline{E}_z}$) the standard deviation of the individual fluctuations δJ and $\delta\overline{E}_z$. Since $\overline{E}_z \propto B$, operating at small magnetic fields can suppress fluctuations of the Zeeman splitting, giving rise to $T_z^* = \frac{1}{\sqrt{2}\sigma_J}$. In contrast to T_x^*, thus, T_z^* is not suppressed at small magnetic fields. This is consistent with the observations in the main text that $T_z^* < T_x^*$.

Two-qubit Hamiltonian

The derivation of the Hamiltonian of two coupled $S-T_-$ qubits is analogous to that of the single-qubit Hamiltonian. The first step is separating the spin and charge degree of freedom, resulting in a generalized Heisenberg Hamiltonian. We give explicit expressions for the two-qubit Hamiltonian for two different architectures, a simplified architecture with single connectivity and the design from the experiment.

Linear chain with a single inter-qubit tunnel (exchange) coupling and isotropic g-tensor up to a scaling. In this case, the multi-qubit Hamiltonian is given by

$$H_{\mathrm{FH}} = \sum_{i=1} \mu_B g_i B S_{z,i} + \sum_{i=1} \frac{\Delta_{ST_-,i}}{\hbar} S_x + \sum_{\langle i,j \rangle} + J_{ij} \left(\frac{\boldsymbol{S}_i \cdot \boldsymbol{S}_j}{\hbar^2} - \frac{1}{4} \right), \tag{20}$$

where $\langle i,j \rangle$ denotes neighboring quantum dots. Projected on the two-qubit basis $\{|S,S\rangle, |S,T_-\rangle, |T_-,S\rangle, |T_-,T_-\rangle\}$ the two-qubit Hamiltonian reads

$$H_{\mathrm{2Q}} = H_{\mathrm{Q1}} + H_{\mathrm{Q2}} + H_{\mathrm{coup,iso}} \tag{21}$$

with

$$H_{\mathrm{Q1}} + H_{\mathrm{Q2}} = \begin{bmatrix} -J_{ij} - J_{kl} & \frac{\Delta_{\mathrm{SO},kl}}{2} & \frac{\Delta_{\mathrm{SO},ij}}{2} & 0 \\ \frac{\Delta_{\mathrm{SO},kl}}{2} & -J_{ij} - \overline{E}_{z,kl} & 0 & \frac{\Delta_{\mathrm{SO},ij}}{2} \\ \frac{\Delta_{\mathrm{SO},ij}}{2} & 0 & -\overline{E}_{z,ij} - J_{kl} & \frac{\Delta_{\mathrm{SO},kl}}{2} \\ 0 & \frac{\Delta_{\mathrm{SO},ij}}{2} & \frac{\Delta_{\mathrm{SO},kl}}{2} & -\overline{E}_{z,ij} - \overline{E}_{z,kl} \end{bmatrix}, \tag{22}$$

$$H_{\mathrm{coup,iso}} = \frac{1}{2} \begin{bmatrix} 0 & 0 & 0 & 0 \\ 0 & 0 & J_{\mathrm{coup},ij,kl} & 0 \\ 0 & J_{\mathrm{coup},ij,kl} & 0 & 0 \\ 0 & 0 & 0 & J_{\mathrm{coup},ij,kl} \end{bmatrix}, \tag{23}$$

Here the subscripts indicate the sites i, j, k and l, respectively. Note that this Hamiltonian is identical to the Hamiltonian used in the main text up to an energy offset that corresponds to a global phase shift.

From a quantum information point of view, the isotropic exchange Hamiltonian (23) generates a universal two-qubit gate. More precisely, the Hamiltonian generates a SWAP + iSWAP gate. This can be visualized through the symmetry of the system: since the isotropic exchange interaction is given by the projector on the singlet subspace, the $|T_-T_-\rangle \langle T_-T_-|$ matrix element of the two-qubit interaction has to be zero.

Ladder with two inter-qubit tunnel (exchange) couplings and anisotropic g-tensors. This is the architecture used in the present device. Consequently, the Hamiltonian cannot be cast into Eq. (20) and an additional rotation of the spin has to be taken into account [13]. Fortunately, the "additional" rotation from the exchange interaction can be absorbed into the rotation caused by the differences in g-tensor for a fixed magnetic field setting. In this case, the total two-qubit Hamiltonian is given by

$$H_{\mathrm{2Q}} = H_{\mathrm{Q1}} + H_{\mathrm{Q2}} + H_{\mathrm{coup,iso}} + H_{\mathrm{coup,SOI}}, \tag{24}$$

where the first three terms are given in Eqs. (22)-(23). The last term is caused by the spin-orbit interaction and is given by

$$H_{\mathrm{coup,SOI}} = P_{\mathrm{2Q}} U_{ij,kl} \left[\left(\frac{\boldsymbol{S}_i \cdot \mathcal{J}_{ik} \boldsymbol{S}_k}{\hbar^2} - \frac{1}{4} \right) + \left(\frac{\boldsymbol{S}_j \cdot \mathcal{J}_{jl} \boldsymbol{S}_l}{\hbar^2} - \frac{1}{4} \right) \right] U^\dagger_{ij,kl} P_{\mathrm{2Q}} - H_{\mathrm{coup,iso}}, \tag{25}$$

$$= \frac{1}{2} \begin{bmatrix} 0 & 0 & 0 & J^{\mathrm{SO}}_{\perp 2,ij,kl} \\ 0 & 0 & J^{\mathrm{SO}}_{\perp 1,ij,kl} & J^{\mathrm{SO}}_{a2,ij,kl} \\ 0 & J^{\mathrm{SO},*}_{\perp 1,ij,kl} & 0 & J^{\mathrm{SO}}_{a1,kl,ij} \\ J^{\mathrm{SO},*}_{\perp 2,ij,kl} & J^{\mathrm{SO},*}_{a2,ij,kl} & J^{\mathrm{SO},*}_{a1,kl,jk} & -J^{\mathrm{SO}}_{||,ij,kl} \end{bmatrix}, \tag{26}$$

where P_{2Q} is the projector on the two-qubit subspace,

$$U_{ij,kl} = U_{\phi_{ij}} U_{\phi_{kl}} e^{i \boldsymbol{v}_{ij} \cdot \boldsymbol{S}^0_{ij}/\hbar} e^{i \boldsymbol{v}_{kl} \cdot \boldsymbol{S}^0_{kl}/\hbar}, \tag{27}$$

is the combination of the single $S - T_-$ qubit basis transformation from the previous section, and $\mathcal{J}_{ik(jl)}$ is the exchange tensor between the spins in quantum dot pair ik (jl) in the spin-orbit basis [13] of quantum dot pair ij and kl. We also note that the full characterization of all elements cannot be resolved with our current operation regime and measurement setup, as high-fidelity sequential spin readout is required. For example, the $J^{\mathrm{SO}}_{\perp 2,ij,kl}$ term could be characterized in the regime where the energies of the $|SS\rangle$ and $|T_-T_-\rangle$ states are identical but energetically separated from the other states. Therefore, we consider for simplicity an isotropic model to describe the swapping dynamics, which fits well to the observations.

Simulation of the two-qubit energy spectrum To compute the two-qubit energy spectrum between Q3 and Q4 in the main text (Fig. 3b), we use the Hamiltonian of (21) and Eq. (7) and the estimated single-qubit parameters at $B =$ 10 mT. Explicitly, we use $U_{Q3} = 290$ GHz, $U_{Q4} = 326$ GHz, $t_{Q3} = 3$ GHz, $t_{Q4} = 2.8$ GHz, $\varepsilon_{Q3} =$ -174 GHz, $\overline{g}_{Q3} =$ 0.37, $\overline{g}_{Q4} = 0.35$, and assume for simplicity a homogeneous $\Delta_{\mathrm{SO},ij} = \Delta_{\mathrm{SO},kl} = 10$ MHz. For the two-qubit interaction between Q3 and Q4, we use $H_{\mathrm{coup,iso}}$ with $J_{\mathrm{coup}} = (J_{ik} + J_{jl})/2$. We have estimated the inter-qubit exchange using $t_{ik} = 2$ GHz and $t_{jl} = 0.4$ GHz and $\varepsilon_{ik} = (\varepsilon_{ij} - \varepsilon_{kl} + \mu_0)/2$ and $\varepsilon_{jl} = (\varepsilon_{kl} - \varepsilon_{ij} + \mu_0)/2$ by assuming the difference between the chemical potential offsets of two qubits $\mu_0 = \mu_{ij} - \mu_{kl} = 0$.

Simulation of the state transfer For the simulation of the state transfer in the main text (Fig. 4b), we use the Hamiltonian of Eq. (23) for the SWAP operations with $J_{\mathrm{coup}} = 95$ MHz and a single-qubit x-axis rotation frequency of Q4 of 13 MHz.

Fitting Hamiltonian Since our two-qubit characterization was performed in a regime where the single-qubit energies are larger than the inter-qubit coupling energies, the upper Hamiltonian can be further simplified using another block-diagonalization (SW approximation) step to ease fitting. In particular, we assume $|J^{\mathrm{SO},*}_{\perp 2,kl,jk}|, |J^{\mathrm{SO},*}_{a1,kl,jk}|, |J^{\mathrm{SO},*}_{a2,kl,jk}| \ll |J_{ij} - \overline{E}_{z,ij}|, |J_{kl} - \overline{E}_{z,kl}|$. The effective Hamiltonian can then be written as follows:

$$H_{\mathrm{2Q,\ eff}} = \frac{J_{\mathrm{trans}}}{4}(\sigma_x^{ij}\sigma_x^{kl} + \sigma_y^{ij}\sigma_y^{kl}) + \frac{J_{\mathrm{cross}}}{4}(\sigma_y^{ij}\sigma_x^{kl} - \sigma_x^{ij}\sigma_y^{kl}) + \frac{J_{\mathrm{long}}}{4}\sigma_z^{ij}\sigma_z^{kl} + \frac{\varepsilon}{4}(\sigma_z^{ij} - \sigma_z^{kl}) + \frac{\Sigma}{4}(\sigma_z^{ij} + \sigma_z^{kl}) \tag{28}$$

with the parameters

$$J_{\mathrm{trans}} = J_{\mathrm{coup},ij,kl} + \mathrm{Re}\left(J^{\mathrm{SO}}_{\perp 1,ij,kl} + \frac{1}{2}\frac{J^{\mathrm{SO},*}_{a2,ij,kl}J^{\mathrm{SO}}_{a1,ij,kl}}{\overline{E}_{z,ij} + \overline{E}_{z,kl}}\right), \tag{29}$$

$$J_{\mathrm{cross}} = -\mathrm{Im}\left(J^{\mathrm{SO}}_{\perp 1,ij,kl} + \frac{1}{2}\frac{J^{\mathrm{SO},*}_{a2,ij,kl}J^{\mathrm{SO}}_{a1,ij,kl}}{\overline{E}_{z,ij} + \overline{E}_{z,kl}}\right) \tag{30}$$

$$J_{\mathrm{long}} = \frac{J_{\mathrm{coup},ij,kl} - J^{\mathrm{SO}}_{||,ij,kl}}{2} - \frac{1}{2}\frac{|J^{\mathrm{SO}}_{a1,ij,kl}|^2 + |J^{\mathrm{SO}}_{a2,ij,kl}|^2}{\overline{E}_{z,ij} + \overline{E}_{z,kl}}, \tag{31}$$

$$\varepsilon = \overline{E}_{z,ij} - \overline{E}_{z,kl} - J_{ij} + J_{kl} + \frac{1}{4}\frac{|J^{\mathrm{SO}}_{a2,ij,kl}|^2 - |J^{\mathrm{SO}}_{a1,ij,kl}|^2}{\overline{E}_{z,ij} + \overline{E}_{z,kl}}, \tag{32}$$

$$\Sigma = \overline{E}_{z,ij} + \overline{E}_{z,kl} - J_{ij} - J_{kl} - \frac{J_{\mathrm{coup},ij,kl} - J^{\mathrm{SO}}_{||,ij,kl}}{2} + \frac{1}{4}\left(\frac{|J^{\mathrm{SO}}_{a1,ij,kl}|^2 + |J^{\mathrm{SO}}_{a2,ij,kl}|^2}{\overline{E}_{z,ij} + \overline{E}_{z,kl}} + \frac{2|J^{\mathrm{SO}}_{\perp 2,ij,kl}|^2}{\overline{E}_{z,ij} + \overline{E}_{z,kl} - J_{ij} - J_{kl}}\right). \tag{33}$$

Here, J_{trans} denotes the real part and J_{cross} the imaginary part of the center off-diagonal terms of the Hamiltonian (24); J_{long} mainly refers to the exchange interaction of $S - T_-$ qubits plus the spin-orbit coupling induced anisotropic exchange interactions; ε includes mainly the detuning of qubit energies in the experiment, and Σ is the total qubit energy, with a correction of the anisotropic exchange couplings. We remark, that the upper Hamiltonian also considers the effect of single-qubit phase rotations that may happen before and after the $\sqrt{\mathrm{SWAP}}$ gate in the GST experiment, e.g., through errors in the idling gate before and after the exchange pulse or crosstalk. Such single-qubit phase rotations shift the $\sqrt{\mathrm{SWAP}}$ rotation axis $\theta \equiv \arg(J_{\mathrm{trans}} + iJ_{\mathrm{cross}}) \rightarrow \theta + \phi$, where arg denotes the argument of the complex number. It can also be noted that this realistic Hamiltonian is similar in form to the isotropic Hamiltonian in the main text up to single-qubit phases and controlled phase rotations, thus we used only the isotropic model to describe the SWAP dynamics in the main text.

VIII. GATE SET TOMOGRAPHY OF THE SINGLE- AND TWO-QUBIT GATE

Gate set tomography of the single-qubit gate

The gate set tomography experiments are all carried out using the python package pyGSTi [19].

For single-qubit GST, we use the model smq1Q_XY with a gateset including only $\sqrt{X}$ and $\sqrt{Y}$. The fiducials for state preparation and measurements are {null, $\sqrt{X}$, $\sqrt{Y}$, $\sqrt{X}\sqrt{X}$, $\sqrt{X}\sqrt{X}\sqrt{X}$, $\sqrt{Y}\sqrt{Y}\sqrt{Y}$}, where null is an idle gate with zero waiting time, and the germs for amplifying qubit errors are {$\sqrt{X}\sqrt{Y}$, $\sqrt{X}\sqrt{X}\sqrt{Y}$}. Unless indicated otherwise, the implemented circuit lengths are powers of two from 1 up to 32, resulting in 568 circuits in total. In every sequence, the singlet or triplet probability of the involved qubit is acquired by averaging over 1000

single-shot cycles. The data was analyzed using pyGSTi with the CPTP (completely positive trace-preserving) model, from which we obtained Pauli transfer matrices (PTM) M_{exp} for each gate operation. By comparing them to the ideal matrices, M_{ideal}, we can derive an entanglement fidelity as $F_p = \text{Tr}[M_{\text{ideal}}^{-1} M_{\text{exp}}]/d^2$, and thus a gate infidelity $1 - F_g = \frac{d}{d+1}(1 - F_p)$, where $d = 2^N$ is the dimension of the Hilbert space, and N refers to the qubit number. Two datasets of the gate infidelities of each qubit are shown in Table I.

It is worth mentioning that in some cases, the $\sqrt{Y}$ gate shows a smaller error than the $\sqrt{X}$ gate, which may be an artefact caused by the GST protocol, i.e., biased attribution of relational errors [20]. Table I also includes the non-unitary gate infidelities, $\frac{d-1}{d}(1 - \sqrt{u(M_{\text{exp}}^{-1} M_{\text{ideal}})}$, where $u(M) = \text{Tr}(J_\alpha(M)^2)$ and $J_\alpha(M)$ is the Jamiołkowski isomorphism map between the matrix M and the corresponding Choi Matrix. Almost all the data show a larger error of the gate infidelity than the non-unitary error, but only by a small amount. Therefore, we can expect slightly higher control fidelities with improved pulse control.

Gate	Q1	Q2	Q3	Q4
$\sqrt{X}$	0.0084±0.0004	0.0039±0.0004	0.0102±0.0009	0.0044±0.0004
$\sqrt{X}$, non-unitary	0.0083±0.0004	0.0038±0.0004	0.0097±0.0009	0.0025±0.0004
$\sqrt{Y}$	0.0062±0.0004	0.0051±0.0005	0.0068±0.0008	0.0055±0.0004
$\sqrt{Y}$, non-unitary	0.0061±0.0004	0.0049±0.0005	0.0060±0.0008	0.0034±0.0004
$\sqrt{X}$	0.0079±0.0004	0.0031±0.0006	0.0100±0.0009	0.0054±0.0015
$\sqrt{X}$, non-unitary	0.0072±0.0004	0.0002±0.0006	0.0093±0.0009	0.0029±0.0015
$\sqrt{Y}$	0.0084±0.0004	0.0114±0.0007	0.0112±0.0009	0.0035±0.0014
$\sqrt{Y}$, non-unitary	0.0075±0.0004	0.0046±0.0007	0.0105±0.0009	0.0010±0.0014

TABLE I. Summary of the gate infidelities of single-qubit gates of Q1-Q4 from two different datasets. The uncertainty represents the 95% confidence interval. Notice the second dataset for Q4 show higher error bars, which was obtained with circuit length L=8, others were obtained with L=32.

Gate set tomography of the two-qubit gate

The two-qubit GST are performed on Q1 and Q2. We derive a model named smq2Q_XYSQRT and the default gateset includes $\{\sqrt{X}_{Q1}, \sqrt{X}_{Q2}, \sqrt{Y}_{Q1}, \sqrt{Y}_{Q2}, \sqrt{\text{SWAP}}\}$, where the subscript in the single-qubit gate refers to the corresponding qubit. The fiducials and germs are shown in Table II. The circuit lengths are powers of two from 1 up to 2, resulting in 1317 circuits in total. In every sequence, the joint probabilities of each qubit are acquired by averaging over 500 single-shot cycles.

	fiducial: preparation	fiducial: measurement	germ
1	null	null	$\sqrt{X}_{Q1}$
2	$\sqrt{X}_{Q2}$	$\sqrt{X}_{Q2}$	$\sqrt{X}_{Q2}$
3	$\sqrt{Y}_{Q2}$	$\sqrt{Y}_{Q2}$	$\sqrt{Y}_{Q1}$
4	$\sqrt{X}_{Q2}\sqrt{X}_{Q2}$	$\sqrt{X}_{Q2}\sqrt{X}_{Q2}$	$\sqrt{Y}_{Q2}$
5	$\sqrt{X}_{Q1}$	$\sqrt{X}_{Q1}$	$\sqrt{\text{SWAP}}$
6	$\sqrt{X}_{Q1}\sqrt{X}_{Q2}$	$\sqrt{Y}_{Q1}$	$\sqrt{\text{SWAP}}\sqrt{X}_{Q2}\sqrt{X}_{Q2}\sqrt{\text{SWAP}}\sqrt{X}_{Q2}\sqrt{X}_{Q1}$
7	$\sqrt{X}_{Q1}\sqrt{Y}_{Q2}$	$\sqrt{X}_{Q1}\sqrt{X}_{Q1}$	$\sqrt{X}_{Q1}\sqrt{Y}_{Q2}\sqrt{Y}_{Q1}\sqrt{Y}_{Q2}\sqrt{Y}_{Q1}$
8	$\sqrt{X}_{Q1}\sqrt{X}_{Q2}\sqrt{X}_{Q2}$	$\sqrt{X}_{Q1}\sqrt{X}_{Q2}$	$\sqrt{X}_{Q2}\sqrt{Y}_{Q2}\sqrt{X}_{Q1}\sqrt{Y}_{Q1}$
9	$\sqrt{Y}_{Q1}$	$\sqrt{X}_{Q1}\sqrt{Y}_{Q2}$	$\sqrt{\text{SWAP}}\sqrt{\text{SWAP}}\sqrt{Y}_{Q2}\sqrt{X}_{Q1}\sqrt{Y}_{Q1}$
10	$\sqrt{Y}_{Q1}\sqrt{X}_{Q2}$	$\sqrt{Y}_{Q1}\sqrt{X}_{Q2}$	$\sqrt{X}_{Q2}\sqrt{Y}_{Q2}\sqrt{Y}_{Q2}$
11	$\sqrt{Y}_{Q1}\sqrt{Y}_{Q2}$	$\sqrt{Y}_{Q1}\sqrt{Y}_{Q2}$	$\sqrt{Y}_{Q2}\sqrt{X}_{Q1}\sqrt{\text{SWAP}}\sqrt{X}_{Q1}$
12	$\sqrt{Y}_{Q1}\sqrt{X}_{Q2}\sqrt{X}_{Q2}$		$\sqrt{\text{SWAP}}\sqrt{X}_{Q1}\sqrt{Y}_{Q1}\sqrt{X}_{Q2}\sqrt{Y}_{Q1}\sqrt{\text{SWAP}}$
13	$\sqrt{X}_{Q1}\sqrt{X}_{Q1}$		$\sqrt{Y}_{Q2}\sqrt{Y}_{Q2}\sqrt{Y}_{Q1}\sqrt{Y}_{Q1}\sqrt{X}_{Q2}\sqrt{X}_{Q2}\sqrt{X}_{Q1}$
14	$\sqrt{X}_{Q1}\sqrt{X}_{Q1}\sqrt{X}_{Q2}$		
15	$\sqrt{X}_{Q1}\sqrt{X}_{Q1}\sqrt{X}_{Q2}$		
16	$\sqrt{X}_{Q1}\sqrt{X}_{Q1}\sqrt{X}_{Q2}\sqrt{X}_{Q2}$		

TABLE II. Fiducials and germs for the two-qubit GST circuits

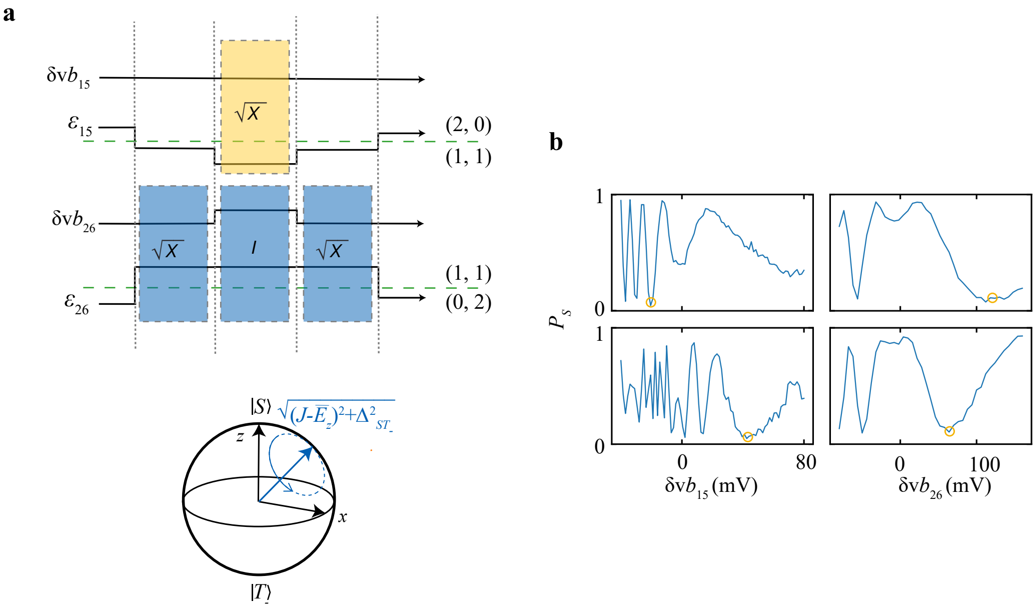

Supplementary Fig 6. **a**, Illustration of the gate voltage pulses for the idle gate calibration of Q2 with Q1 doing an $\sqrt{X}$ gate operation. In this Ramsey-like sequence, a perfect idle gate will result in a zero singlet probability of Q2. The Bloch sphere below shows the qubit rotation with a barrier gate pulse. **b**, Measured singlet probabilities P_S as a function of the barrier voltage change with a fixed duration that equals the operation time on the other qubit. They are the results for the calibration of $\sqrt{X}_{Q2}$ (top left), $\sqrt{X}_{Q1}$ (top right), $\sqrt{Y}_{Q2}$ (bottom left) and $\sqrt{Y}_{Q1}$ (bottom right). The orange circles show the expected position of the barrier gate voltage for the idle operation.

As discussed in the Extended Fig. 10 in the main text, the single-qubit gate in the two-qubit space requires an idle gate. Our strategy is to pulse the idle qubit to an operating point where it completes a 2π rotation during the time needed to operate on the other qubit, with a rotation speed of $f_{ST_-} = \sqrt{(J - \overline{E}_z)^2 + \Delta^2_{ST_-}}/h$ by pulsing the barrier gate. To calibrate the barrier pulse, we use a Ramsey-like pulse sequence as shown in Supplementary Fig. 6a. One example of the barrier pulse calibration for the single-qubit gates of Q1 and Q2 is shown in Supplementary Fig. 6b. Here, we keep the idle qubit at the sweet point of the detuning and try to pulse the barrier gate positively to reduce the effect of charge noise from strong exchange coupling. For $\sqrt{X}_{Q1}$, $\sqrt{Y}_{Q1}$, $\sqrt{Y}_{Q2}$ the idling qubit is pulsed positively, while for $\sqrt{X}_{Q2}$, the idling qubit is pulsed negatively because the short $\sqrt{X}$ gate duration of Q2 otherwise does not allow a full rotation on the idling qubit (Q1) given the accessible values of $|\overline{E}_z - J|$.

The raw data of running GST circuits was analyzed using the CPTP model, and the gate infidelities of three datasets are shown in Table. III. The single-qubit gate errors in the two-qubit space are quite large compared to those measured in the single-qubit space. To get a better understanding, we calculate the Jamiołkowski probability $\epsilon_J(\mathcal{L}) = -\mathrm{Tr}[\rho_J(\mathcal{L}) \left|\Psi\right\rangle \left\langle\Psi\right|]$ and the Jamiołkowski amplitude $\theta_J(\mathcal{L}) = ||(1 - \left|\Psi\right\rangle \left\langle\Psi\right|)\rho_J(\mathcal{L}) \left|\Psi\right\rangle||$, which approximately describe the amount of incoherent and coherent Hamiltonian errors of the quantum process, respectively [21]. Here $\rho_J(\mathcal{L}) = (\mathcal{L} \otimes 1_{d^2}) \left|\Psi\right\rangle \left\langle\Psi\right|$ is the Jamiołkowski state, $\mathcal{L} = \log(M_{\mathrm{exp}} M^{-1}_{\mathrm{ideal}})$ is the error generator of the process, and $\left|\Psi\right\rangle$ is a maximally entangled state that originates from the relation of quantum processes to states in a Hilbert space twice the dimension via the Choi-Jamiołkowski isomorphism. In Table. III, we can see that the coherent errors are all larger than the incoherent errors, suggesting the qubits suffer more from calibration errors in the gate pulses rather than decoherence. These calibration errors affect not only the qubit we aim to rotate but also the idling qubit, which is ideally undergoing a 2π rotation. The total error they contribute to the gate operation can be approximately calculated as $1 - F_J = \frac{d}{d+1}[\epsilon_J(\mathcal{L}) + \theta_J(\mathcal{L})^2]$ when errors are small. We can observe that they are similar to the values $1 - F_g$ calculated before.

For the two-qubit gate, the GST model we used for data analysis is a standard $\sqrt{\mathrm{SWAP}}$. From the GST result, we obtain a PTM and fit it to our theoretical model (28). The fitted parameters are shown in Table IV. The fact that J_{long} differs from J_{trans}, as well as the non-zero value of J_{cross}, arise from spin-orbit coupling. The detuning of two qubit splittings ε can originate from imperfect calibration of the $\sqrt{\mathrm{SWAP}}$ gate operation point, whose strength compared to the exchange coupling is calculated and shown as $\varepsilon / \sqrt{J^2_{\mathrm{trans}} + J^2_{\mathrm{cross}}}$. Σ is a single-qubit z rotation, see

Gate	Gate infidelity (1-F_g)	Jamiołkowski probability $\epsilon_J(\mathcal{L})$	Jamiołkowski amplitude $\theta_J(\mathcal{L})$	Total error (1-F_J)
$\sqrt{X}_{Q1}$	0.02826	0.01827	0.1337	0.02891
$\sqrt{X}_{Q2}$	0.03796	0.04513	0.06360	0.03934
$\sqrt{Y}_{Q1}$	0.04175	0.03593	0.13510	0.04335
$\sqrt{Y}_{Q2}$	0.13356	0.10504	0.28204	0.14767
$\sqrt{\text{SWAP}}$	0.20320	0.25753	0.22752	0.24743
$\sqrt{X}_{Q1}$	0.02535	0.02029	0.11011	0.02593
$\sqrt{X}_{Q2}$	0.04334	0.05166	0.06701	0.04492
$\sqrt{Y}_{Q1}$	0.04078	0.04056	0.11085	0.04228
$\sqrt{Y}_{Q2}$	0.13500	0.10932	0.27864	0.14957
$\sqrt{\text{SWAP}}$	0.20390	0.25132	0.23363	0.24472
$\sqrt{X}_{Q1}$	0.03027	0.02342	0.12412	0.03106
$\sqrt{X}_{Q2}$	0.04032	0.04576	0.08005	0.04173
$\sqrt{Y}_{Q1}$	0.04232	0.04027	0.12052	0.04384
$\sqrt{Y}_{Q2}$	0.13230	0.10893	0.27269	0.14663
$\sqrt{\text{SWAP}}$	0.19560	0.24216	0.22064	0.23267

TABLE III. Summary of the gate infidelities of the two-qubit GST measurement from three different datasets. Notice the fidelities for the $\sqrt{\text{SWAP}}$ is analyzed by fitting the theoretical model.

Eq. (28). With those fitted parameters, we can rebuild the Pauli transfer matrix M_{ideal} of the $\sqrt{\text{SWAP}}$ gate, and by comparing it to the experimental result M_{exp}, we obtain the gate fidelities as shown in the Table IV. These gate fidelities are lower than the Bell state fidelity, which possibly may be explained by the fact that the gate fidelity is a measure that averages over all possible input states, while the Bell state fidelity considers only one specific input state. Moreover, the pulse schemes for measuring the $\sqrt{\text{SWAP}}$ gate fidelity and the Bell state are different, which can result in different contributions from single-qubit errors. These fitted parameters also enable us to rebuild the two-qubit gate unitary matrix as shown below (using data set 3 in Table. IV):

$$\sqrt{\text{SWAP}}_{ST} = \begin{pmatrix} 1 & 0 & 0 & 0 \\ 0 & 0.748e^{3.131i} & 0.664e^{-1.45i} & 0 \\ 0 & 0.664e^{-1.95i} & 0.748e^{2.894i} & 0 \\ 0 & 0 & 0 & 1e^{0.266i} \end{pmatrix}, \tag{34}$$

Parameter	Data set 1	Data set 2	Data set 3
$J_{\text{trans}}t$ (rad)	4.313	4.528	4.624
$J_{\text{cross}}t$ (rad)	-1.187	-1.285	-1.181
$J_{\text{long}}t$ (rad)	-0.465	-0.531	-0.524
εt (rad)	1.540	1.148	0.635
$\sum t$ (rad)	0.190	0.218	0.266
$\varepsilon/\sqrt{J_{\text{trans}}^2 + J_{\text{cross}}^2}$	0.344	0.244	0.133
Gate Fidelity	79.7%	79.6%	80.4%

TABLE IV. Summary of the fitting parameters for the $\sqrt{\text{SWAP}}_{ST}$ gate. In the table, t refers to the operation time of the $\sqrt{\text{SWAP}}_{ST}$ gate. The values of the parameters with a radiation unit are modulo 2π.

[1] Hendrickx, N. *et al.* A single-hole spin qubit. *Nat. Commun.* **11**, 3478 (2020).

[2] Hsiao, T.-K. *et al.* Exciton transport in a germanium quantum dot ladder. *Phys. Rev. X* **14**, 011048 (2024).

[3] Wang, C.-A. *et al.* Probing resonating valence bonds on a programmable germanium quantum simulator. *npj Quantum Inf.* **9**, 58 (2023).

[4] Nichol, J. M. *et al.* Quenching of dynamic nuclear polarization by spin-orbit coupling in GaAs quantum dots. *Nat. Commun.* **6**, 7682 (2015).

[5] Jirovec, D. *et al.* Dynamics of hole singlet-triplet qubits with large g-factor differences. *Phys. Rev. Lett.* **128**, 126803 (2022).

[6] Hendrickx, N. *et al.* Sweet-spot operation of a germanium hole spin qubit with highly anisotropic noise sensitivity. *Nature Materials* 1–8 (2024).

[7] Hu, X. & Das Sarma, S. Charge-fluctuation-induced dephasing of exchange-coupled spin qubits. *Phys. Rev. Lett.* **96**, 100501 (2006).

[8] Huang, P., Zimmerman, N. M. & Bryant, G. W. Spin decoherence in a two-qubit CPHASE gate: the critical role of tunneling noise. *npj Quantum Inf.* **4**, 62 (2018).

[9] Shulman, M. D. *et al.* Demonstration of entanglement of electrostatically coupled singlet-triplet qubits. *Science* **336**, 202–205 (2012).

[10] Nichol, J. M. *et al.* High-fidelity entangling gate for double-quantum-dot spin qubits. *npj Quantum Inf.* **3**, 3 (2017).

[11] Burkard, G., Ladd, T. D., Pan, A., Nichol, J. M. & Petta, J. R. Semiconductor spin qubits. *Rev. Mod. Phys.* **95**, 025003 (2023).

[12] Danon, J. & Nazarov, Y. V. Pauli spin blockade in the presence of strong spin-orbit coupling. *Phys. Rev. B* **80**, 041301 (2009).

[13] Geyer, S. *et al.* Anisotropic exchange interaction of two hole-spin qubits. *Nature Physics* 1–6 (2024).

[14] Burkard, G., Loss, D. & DiVincenzo, D. P. Coupled quantum dots as quantum gates. *Phys. Rev. B* **59**, 2070–2078 (1999).

[15] Jirovec, D. *et al.* A singlet-triplet hole spin qubit in planar Ge. *Nat. Mater.* **20**, 1106–1112 (2021).

[16] Bravyi, S., Divincenzo, D. P. & Loss, D. Schrieffer-Wolff transformation for quantum many-body systems. *Annals of Physics* **326**, 2793 (2011).

[17] Chirolli, L. & Burkard, G. Decoherence in solid-state qubits. *Advances in Physics* **57**, 225–285 (2008).

[18] Lawrie, W. I. L. *et al.* Simultaneous single-qubit driving of semiconductor spin qubits at the fault-tolerant threshold. *Nature Communications* **14**, 3617 (2023).

[19] Nielsen, E. *et al.* Probing quantum processor performance with pygsti. *Quantum science and Technology* **5**, 044002 (2020).

[20] Mądzik, M. T. *et al.* Precision tomography of a three-qubit donor quantum processor in silicon. *Nature* **601**, 348–353 (2022).

[21] Blume-Kohout, R. *et al.* A taxonomy of small markovian errors. *PRX Quantum* **3**, 020335 (2022).
